# Dihydrocaffeic Acid—Is It the Less Known but Equally Valuable Phenolic Acid?

**DOI:** 10.3390/biom13050859

**Published:** 2023-05-18

**Authors:** Bartłomiej Zieniuk

**Affiliations:** Department of Chemistry, Institute of Food Sciences, Warsaw University of Life Sciences—SGGW, 159c Nowoursynowska St., 02-776 Warsaw, Poland; bartlomiej_zieniuk@sggw.edu.pl; Tel.: +48-22-59-37-621

**Keywords:** dihydrocaffeic acid, phenolic acids, antioxidants, biological properties, enzymatic synthesis, lipases, kukoamines

## Abstract

Dihydrocaffeic acid (DHCA) is a phenolic acid bearing a catechol ring and three-carbon side chain. Despite its being found in minor amounts in numerous plants and fungi of different origins, it has attracted the interest of various research groups in many fields of science, from food to biomedical applications. The review article presented herein aims to show a wider audience the health benefits and therapeutic, industrial, and nutritional potential of dihydrocaffeic acid, by sheddinglight on its occurrence, biosynthesis, bioavailability, and metabolism. The scientific literature describes at least 70 different derivatives of dihydrocaffeic acid, both those occurring naturally and those obtained via chemical and enzymatic methods. Among the most frequently used enzymes that were applied for the modification of the parent DHCA structure, there are lipases that allow for obtaining esters and phenolidips, tyrosinases used for the formation of the catechol ring, and laccases to functionalize this phenolic acid. In many studies, both in vitro and in vivo, the protective effect of DHCA and its derivatives on cells subjected to oxidative stress and inflammation were acknowledged.

## 1. Introduction

**Figure 1:**
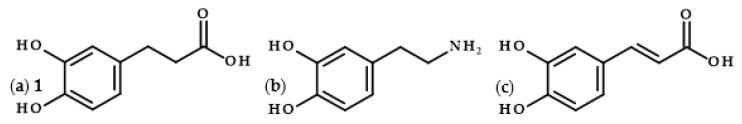
Chemical structures of dihydrocaffeic acid **1** (**a**), dopamine (**b**), and caffeic acid (**c**).

Dihydrocaffeic acid (3-(3,4-dihydroxyphenyl)propanoic acid, DHCA **1**; Figure 1a) is a phenolic acid belonging to the group of phenylpropanoic acids, which can be differentiated by a six-carbon aromatic ring and a three-carbon side chain with the carboxyl group at the end of the carbon chain. The molecule of DHCA itself comprises catechol moiety and the aforementioned propanoic tail. In some ways, it also resembles the structure of dopamine (2-(3,4-dihydroxyphenyl)ethylamine, Figure 1b) [1], one of the significant neuromodulatory molecules. From the physical-chemical point of view, dihydrocaffeic acid is a white to beige to orange powder. It has a molecular weight of 182.17 g/mol and a melting point of 136 °C. It is soluble in water and ethanol and has very limited solubility in nonpolar organic solvents [2]. DHCA is a phytochemical that occurs naturally in a number of plants, but it is definitely present less frequently and in lesser amounts in comparison with its unsaturated derivative, i.e., caffeic acid (Figure 1c).

The review article presented herein intends to bring the scientific community’s attention to the health benefits and therapeutic, industrial, and nutritional potential of dihydrocaffeic acid. The following issues related to this phenolic acid were raised: the occurrence of DHCA and its derivatives, health benefits and biological activities, its formation and metabolism, and the recent advances in its (chemo)enzymatic modifications.

## 2. The Occurrence of Dihydrocaffeic Acid and Its Derivatives

The presence of dihydrocaffeic acid has been confirmed in various plant species, including fruits, lycophytes, and ornamental and medicinal plants [3,4,5,6,7,8,9,10,11,12,13,14,15,16,17,18], and the available data are summarized in Table 1. DHCA with a concentration of 0.03 mg/kg dry weight was a minor constituent determined to be in the methanolic extract of dyer’s woad leaves (*Isatis tinctoria*), a plant belonging to the Brassicaceae family and with a long history of use both in folk medicine and for the extraction of indigo dye from the leaves [3]. Phenolic acid, of interest, has also been confirmed as an ingredient of extracts of two Asteraceae plants, namely in the white ray florets of *Matricaria recutita* [4] and in the fresh aerial parts of *Gynura bicolor* [5]. It is one of the most numerous families of vascular plants; therefore, the statement that DHCA is present in plants of the Asteraceae family is an extremely exaggerated statement. Moreover, dihydrocaffeic acid was one of the 35 constituents of transformed root cultures of *Nepeta teydea*, a plant from the Lamiaceae family. The freeze-dried hairy roots of *N. teydea* were extracted with methanol, and individual fractions were then purified to give 2 mg of DHCA (10.53 mg/kg freeze-dried roots) [6]. Other natural sources of dihydrocaffeic acid are: the aerial part of *Lindera glauca* (0.306 mg/kg dry weight) [7], the whole grasses of *Selaginella stautoniana* [8], the flowers of the Australian rainforest tree *Polyscias murrayi*—with the highest observed concentration in the literature of 352.32 mg/kg fresh weight [9], the concentrated juice of *Rosa roxburghii* (0.3 g/L) [10], and *Melipona beecheii* Cuban polyfloral honey [11].

Furthermore, the presence of this acid was claimed in four different varieties of dates, namely Tantbouchte, Tafizaouine, Tazerzait, and Tazizaout [12]. Going further, olives, especially, but not only, black ones, as well as olive oils, are a known source of phenolic compounds, and, therefore, DHCA [13,14,15]. Black olives pericarp was an abundant source of phenolic compounds and dihydrocaffeic acid, with a determined content of 1.790 ± 0.030 g/kg dry weight, was the fourth most significant compound after hydroxytyrosol, acetoside-1, and acetoside-2. In olive processing, brining is a method for removing bitterness and increasing the taste of olive drupes, but, simultaneously, may be a process of partially decreasing phenolic content. Therefore, the authors also evaluated the brine in terms of these compounds. The brine consisted mainly of hydroxytyrosol (0.600 ± 0.010 g/L) and dihydrocaffeic acid (0.183 ± 0.001 g/L). On the contrary, green olives and their brine were only a source of hydroxytyrosol, and only traces of other phenolics were detected [13]. Research conducted by Bianco and Uccella [14] on the phenolic compounds of Greek olive varieties (Black Thasos and Black Conservolia cultivars) allows for the conclusion that dihydrocaffeic acid constitutes between 5 and 10% of all biophenolic components. Moreover, the authors validated different procedures for phenolics determination in olive varieties of different country origins. In the Green Douro (Portugal) and the Black Cassanese (Italy) olive cultivars, DHCA was found after hydrolysis with 1M NaOH, suggesting that this acid is a part of more complex structures [14]. An approach of multivariate discriminant analysis and artificial neural networks for the authenticity confirmation of Taggiasca Ligure extra-virgin olive oil was employed by Senizza et al. [15]. The authentic Italian Taggiasca olive oils in comparison with other olive oils were characterized as more abundant in phenolic acids, such as caffeic and dihydrocaffeic acids and other low-molecular phenolics, and the proposed methods allowed for easier authentication and fraud uncovering [15].

It is a well-known fact that alcoholic beverages, e.g., wines, beers, and ciders, are also precious sources of phenolic compounds. The presence of dihydrocaffeic acid was observed in the red wine Lacrima di Morro d’Alba, produced in the region of Marche in Italy [16]. The phenolic profiles of a large group of Asturian (Spain) ciders were analyzed by Madrera et al. [17] and Suarez et al. [18]. For both papers, DHCA was the most abundant and accounted for 12–35% of all phenolic compounds. In the former, 92 natural ciders available in the market from the years 1999 and 2000 were compared, and the content of this acid was in the range from roughly 26 to almost 150 mg/L. The second had a similar concentration range (55.8–110.5 mg/L). Furthermore, the authors suggested that DHCA was generated by the hydrolysis of chlorogenic acid and caffeic acid reduction, concentrations of which were much larger in the apple musts. Lactic acid bacteria present during fermentation or post-fermentation steps (malolactic fermentation) were probably responsible for this biotransformation [17,18]. The matter of the bacterial metabolism of phenolic compounds leading to the formation of dihydrocaffeic acid will be discussed in more detail in Section 4, Metabolism of Dihydrocaffeic Acid by Intestinal and Lactic Acid Bacteria.

DHCA, similarly to other phenolics, i.e., hydroxycinnamic and hydroxypropanoic acids, often does not occur in free form, and its derivatives, e.g., in the form of esters and amides, are much more often observed. The data on the derivatives of dihydrocaffeic acid, and their sources and concentrations [5,19,20,21,22,23,24,25,26,27,28,29,30,31,32,33], are summarized in Table 2.

**Figure 2:**
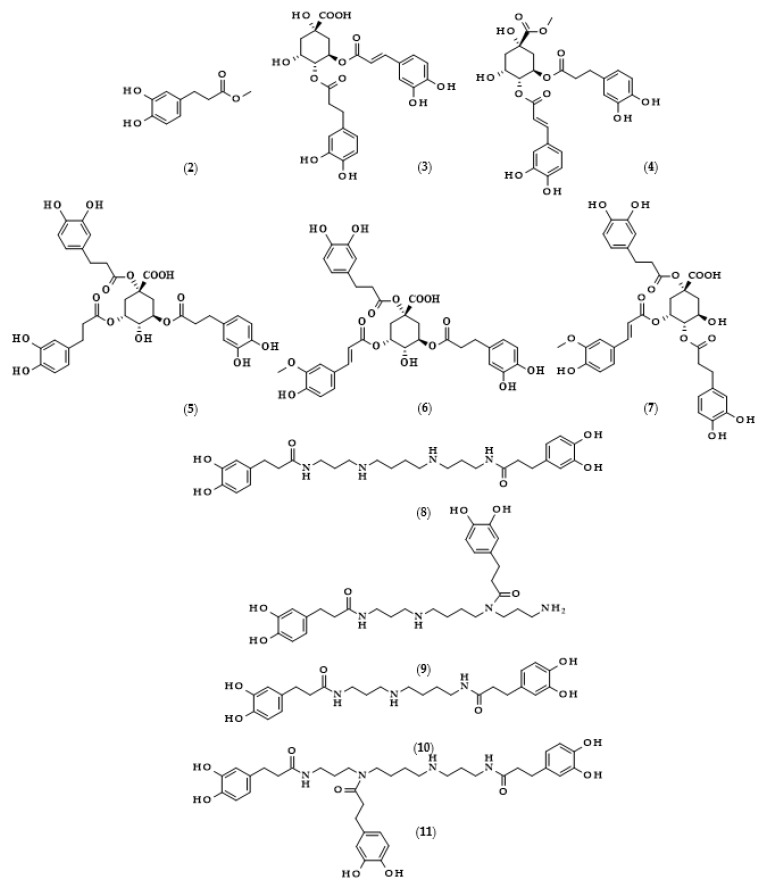

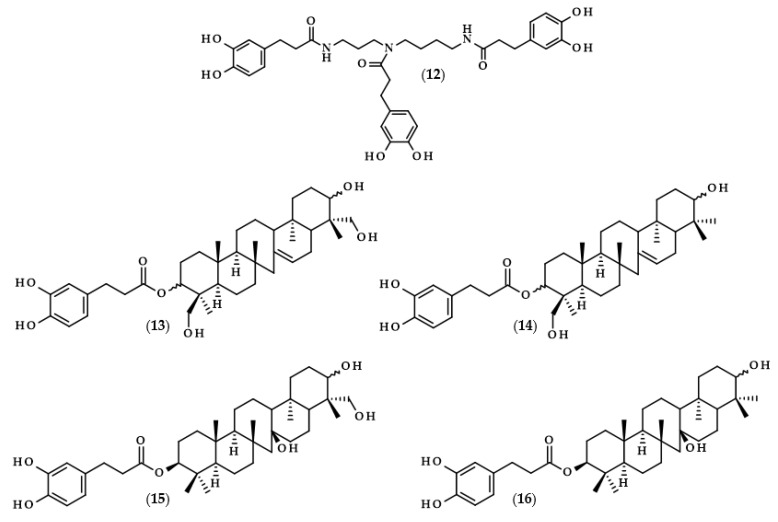
Chemical structures of derivatives of dihydrocaffeic acid occurring in nature. Chemical structures of: methyl dihydrocaffeate (**2**), Tungtungmadic acid (3-caffeoyl-4-dihydrocaffeoyl quinic acid) (**3**), Salicornate (methyl 4-caffeoyl-3-dihydrocaffeoyl quinate) (**4**), Podospermic acid (1,3,5-tris(dihydrocaffeoyl)quinic acid) (**5**), Feruloylpodospermic acid A (1,5-bis(dihydrocaffeoyl)-3-feruloyl quinic acid) (**6**), Feruloylpodospermic acid B (1,4-bis(dihydrocaffeoyl)-3-feruloyl quinic acid) (**7**), Kukoamine A (N^1^,N^12^-*bis*(dihydrocaffeoyl)spermine) (**8**), Kukoamine B (N^1^,N^8^-*bis*(dihydrocaffeoyl)spermine) (**9**) N^1^,N^8^-*bis*(dihydrocaffeoyl)spermidine (**10**), N^1^,N^4^,N^12^-*tris*(dihydrocaffeoyl)spermine (**11**), N^1^,N^4^,N^8^-*tris*(dihydrocaffeoyl)spermidine (**12**), (3α,21β)-Lycophlegmariol A (**13**), (3β,21β)-Lycophlegmariol B (**14**), (3β,21α)-Lycophlegmariol D (**15**), and (3β,21α)-Lycophlegmarin (**16**).

Moreover, the chemical structures of the described derivatives are shown in Figure 2. Methyl dihydrocaffeate (**2**) was isolated along with its carboxylic form from stems of *G. bicolor*, and after undergoing processes of purification, an ester content of 45.45 mg/kg of the ethyl acetate fraction was obtained [5].

Hexose and quinic acid derivatives of the acid of interest are quite common in the literature. Dihydrocaffeic acid hexose isomers were found in the three types of lettuce, namely butterhead, iceberg, and romaine; moreover, Yang et al. [19] elaborated metabolite libraries of these types of lettuce. GC×GC-TOF/MS and UPLC-IMS-QTOF/MS allowed for the detection of three different isomers with the observed masses (*m*/*z*) of 343.1029, 343.1032, and 343.1038. In the comparison between head types of lettuce, it was revealed that the butterhead type had the highest content of phenolics, including dihydroxybenzoic acid and dihydrocaffeic acid hexose isomers [19].

Glasswort (*Salicornia herbacea* L.) is a plant which is similar to horsetail, with fleshy shoots that are widely consumed in Asia and Europe. It is characterized by its salty and slightly grassy taste, and often appears in fish and seafood dishes. Glasswort is considered to be a health-promoting vegetable and several properties and activities of this plant have been confirmed [20]. The rich polyphenolic profile is a source of health benefits, and the derivatives of dihydrocaffeic acid are also found within its composition. The content of the DHCA esterified with quinic acid varied dependent on the maturity stage and was in the range of 36.6–85.1 mg/100 g dry weight [20]. More precisely, the structure of the ester of these phenolic and quinic acids was investigated by Hwang et al. [21], Chung et al. [22], and Chung et al. [23], and the compound is called tungtungmadic acid (3-caffeoyl-4-dihydrocaffeoyl quinic acid) (**3**). As can be seen in Figure 2, this compound is an ester of quinic acid with caffeic acid at position C3 and dihydrocaffeic acid at C4, and its common name came from “Tungtungmadi,” a Korean name for glasswort [22]. In both studies, i.e., [21,23], the authors, after purification processes, obtained 24 mg of tungtungmadic acid from 3 kg of air-dried glasswort. Additionally, Kim et al. [24], besides tungtungmadic acid (0.5125 mg/kg fresh weight), also isolated a novel compound, a methyl ester of the isomer of tungtungmadic acid which has been named salicornate (methyl 4-caffeoyl-3-dihydrocaffeoyl quinate, (**4**); its concentration was quite similar and it was 0.5625 mg/kg fresh weight.

Even more complex chemical structures were elucidated by conducting spectroscopic and mass spectrometry studies of the extracts from *Podospermum laciniatum* (synonym: *Scorzonera laciniata* L.) [25] or the aerial parts of the Mongolian medicinal plant *Scorzonera divaricata* [26]. The compound from the first paper was 1,3,5-tris(dihydrocaffeoyl)quinic acid (podospermic acid, **5**) with a yield of 358.10 mg/kg fresh weight [25]. Isolated compounds from the studies of Tsevegsuren et al. [26], namely feruloylpodospermic acid A (**6**) and feruloylpodospermic acid B (**7**), were isomers and possessed two moieties of dihydrocaffeic acid and one from ferulic acid, and differed only in the positions of the ester group for one of the acids (1,5-bis(dihydrocaffeoyl)-3-feruloyl quinic acid vs. 1,4-bis(dihydrocaffeoyl)-3-feruloyl quinic acid). The content of isomer A in *S. divaricata* was almost four times higher (82.03 mg/kg dry weight) in comparison with isomer B (23.44 mg/kg dry weight).

Kukoamines are another group of dihydrocaffeic acid derivatives. Chemically, they are amides of dihydrocaffeic acid and spermine or spermidine, organic chemical compounds belonging to polyamines. The sources of kukoamines are primarily nightshade plants (Solanaceae); as well, the presence of kukoamine A (N^1^,N^12^-*bis*(dihydrocaffeoyl)spermine, **8**) was confirmed in the stems of Enoki mushrooms (*Flammulina velutipes*) [27,28,29,30,31]. The Kukoamine A structure was revealed in 1980 by Funayama et al. [27], and this amide was found in the root barks of *Lycium chinense*, a well-known oriental medicine called “jikoppi” which has hypotensive, hypoglycemic, and antipyretic activities. A combination of spectroscopic methods and acid hydrolysis afforded the finding of another structure of such amides, kukoamine B (N^1^,N^8^-*bis*(dihydrocaffeoyl)spermine, **9**). Extraction and chromatography allowed for the isolation of 120.7 mg of kukoamine B from 10 kg of the dried root bark of *L. chinense* [28]. Studies on dihydrocaffeoyl polyamines in nightshade plants have also been conducted by Parr et al. [29]. The presence of kukoamines was confirmed in the tubers of 24 different potato varieties. Interestingly, the authors, besides kukoamine A, also described other polyamines, both with spermine and spermidine, with double or triple amide combinations of dihydrocaffeic acid. These were the following chemical compounds: N^1^,N^8^-*bis*(dihydrocaffeoyl)spermidine (**10**), N^1^,N^4^,N^12^-*tris*(dihydrocaffeoyl)spermine (**11**), and N^1^,N^4^,N^8^-*tris*(dihydrocaffeoyl)spermidine (**12**), and their contents in potato tubers were dependent on the varieties. Furthermore, in the other nightshade species, kukoamines were also present, i.e., in the leaf extract of *Nicotiana sylvestris* (probably compounds **8** and **10**), as well as in tomato (*Lycopersicon esculentum*)—low levels of **8**, **10**, and **11** [29]. Forero et al. [31] identified four compounds that are responsible for the bitterness of Lulo (*S. quitoense*) fruits, especially the bitterness that increased during fruit pulp juicing and drying. Two of them were representatives of kukoamine derivatives, namely N^1^,N^8^-*bis*(dihydrocaffeoyl)spermidine (**10**) and N^1^,N^4^,N^8^-*tris*(dihydrocaffeoyl)spermidine (**12**), where the concentration of the latter was approximately 25 mg/kg of fruit pulp [31].

Lycophytes are one of the oldest living clades within vascular plants. They are also a known source of dihydrocaffeic acid and its derivatives, such as in the aforementioned *S. stautoniana* or, e.g., *Huperzia phlegmaria* (synonym: *Lycopodium phlegmaria* L.) [32,33]. The latter among their bioactive compounds contain so-called serratene triterpenoids. Chemically, they are composed of five fused rings, namely three six-carbon cyclohexane rings, one cyclohexene ring (a double bond between C14 and C15), and one seven-member ring. Moreover, they possess seven, mainly methyl, substituents and two hydroxyl groups (at C3, which is often esterified with dihydrocaffeic acid, and at C21) [32]. Wittayalai et al. [32] isolated eight compounds from the methanol extract of *L. phlegmaria*, two pentacyclic triterpenoids, five serratene triterpenoids, and one abietane-type diterpene. In more detail, four triterpenoids were esterified with DHCA, (3α,21β)-Lycophlegmariol A (**13**), (3β,21β)-Lycophlegmariol B (**14**), (3β,21α)-Lycophlegmariol D (**15**), and (3β,21α)-Lycophlegmarin (**16**). The first three compounds were isolated for the first time and their chemical structures were thoroughly elucidated. (3β,21α)-Lycophlegmariol D (**15**) was isolated with the highest yield, i.e., 29.54 mg/kg dry weight, and in the case of the other it ranged from 2.64 to 5.17 mg/kg dry weight [32]. In the research of Nguyen et al. [33], (3α,21β)-Lycophlegmariol A was also isolated as one of the obtained compounds from the same plant, and 10.00 mg/kg dry weight was afforded.

## 3. Biosynthesis of Dihydrocaffeic Acid

**Figure 3:**
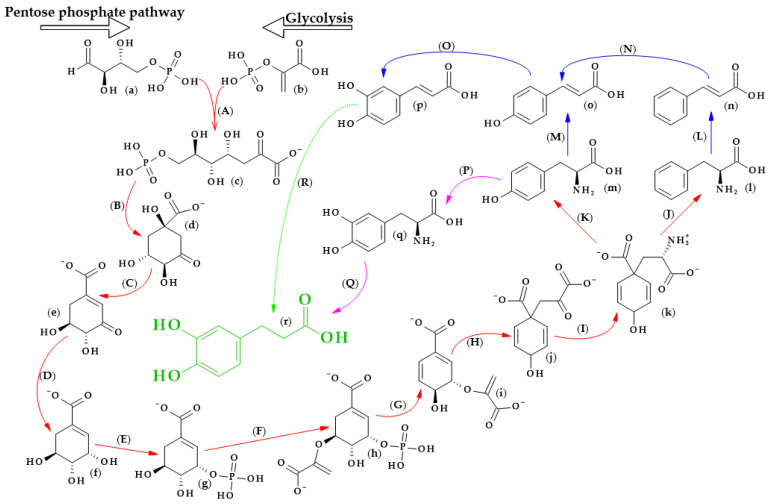
Dihydrocaffeic acid biosynthesis—proposed simplified pathway (adapted and modified from [34,35,36,37,38,39]). Explanations: (**a**)–(**r**)—compounds, A–R—enzymes, i.e., (**a**)—D-Erythrose 4-phosphate, (**b**)—Phosphoenolpyruvate, (**c**)—7-phospho-2-dehydro-3-deoxy-D-arabino-heptonate, (**d**)—3-Dehydroquinate, (**e**)—3-Dehydroshikimate, (**f**)—Shikimate, (**g**)—Shikimate-3-phosphate, (**h**)—5-*O*-(1-carboxyvinyl)-3-phosphoshikimate, (**i**)—Chorismate, (**j**)—Prephenate, (**k**)—L-Arogenate, (**l**)—Phenylalanine, (**m**)—Tyrosine, (**n**)—*trans*-Cinnamic acid, (**o**)—*p*-Coumaric acid, (**p**)—Caffeic acid, (**q**)—3,4-Dihydroxy-L-phenylalanine (L-Dopa), (**r**)—Dihydrocaffeic acid, (**A**)—3-deoxy-7-phosphoheptulonate synthase (EC 2.5.1.54), (**B**)—3-dehydroquinate synthase (EC 4.2.3.4), (**C**)—3-dehydroquinate dehydratase I (EC 4.2.1.10), (**D**)—shikimate dehydrogenase (EC 1.1.1.25), (**E**)—shikimate kinase (EC 2.7.1.71), (**F**)—3-phosphoshikimate 1-carboxyvinyltransferase (EC 2.5.1.19), (**G**)—chorismate synthase (EC 4.2.3.5), (**H**)—chorismate mutase (EC 5.4.99.5), (**I**)—bifunctional aspartate aminotransferase and glutamate/aspartate-prephenate aminotransferase (EC 2.6.1.1, EC 2.6.1.78, EC 2.6.1.79), (**J**)—arogenate/prephenate dehydratase (EC 4.2.1.91, EC 4.2.1.51), (**K**)—arogenate dehydrogenase (NADP^+^) (EC 1.3.1.78), (**L**)—phenylalanine ammonia-lyase (EC 4.3.1.24), (**M**)—phenylalanine/tyrosine ammonia-lyase (EC 4.3.1.25), (**N**)—*trans*-cinnamate 4-monooxygenase (EC 1.14.14.91), (**O**)—*p*-coumarate 3-hydroxylase (EC 1.14.13.-), (**P**)—tyrosine 3-monooxygenase (EC 1.14.16.2), (**Q**)—3,4-dihydroxy-L-phenylalanine ammonia-lyase (EC 4.3.1.22), and (**R**)—double bond reductase (EC 1.3.1.-).

The biosynthesis of dihydrocaffeic acid is a complex process. Unfortunately, the detailed metabolic route for DHCA formation has not been described so far. Referring to [34,35,36,37,38,39], the simplified pathway of dihydrocaffeic acid biosynthesis is presented in Figure 3.

As with other phenolic acids, several common elements of these pathways can be distinguished. It all starts with the formation of 7-phospho-2-dehydro-3-deoxy-D-arabino-heptonate from D-erythrose 4-phosphate and phosphoenolpyruvate, products of the pentose phosphate pathway and glycolysis, respectively. The part of the process in Figure 3 that is marked with red lines is the so-called Shikimate pathway, and in plants it is responsible for the biosynthesis of the aromatic amino acids (phenylalanine, tryptophan, and tyrosine). Eight subsequent reactions lead to obtaining L-arogenate, the last compound in the formation of phenylalanine and its hydroxylated derivative, i.e., tyrosine, and two crucial amino acids in phenylpropanoids biosynthesis [35]. 

Probably two of the most important enzymes for the production of phenolic compounds are phenylalanine ammonia-lyase (EC 4.3.1.24) and phenylalanine/tyrosine ammonia-lyase (EC 4.3.1.25), which allow for obtaining cinnamic acid and *p*-coumaric acid from phenylalanine and tyrosine, respectively. Then, other cinnamic acid derivatives, i.e., caffeic, ferulic, and sinapic acids, are biosynthesized by the action of several hydroxylases, monooxygenases, and methyltransferases [34,36].

Furthermore, by observing the metabolism of tyrosine, it can be seen that tyrosine 3-monooxygenase (EC 1.14.16.2) changes tyrosine to 3,4-dihydroxy-L-phenylalanine (L-Dopa), which, shortly afterwards, can be metabolized to DHCA by another ammonia-lyase (EC 4.3.1.22) [37]. L-Dopa is found in plants of the Fabaceae family, namely, in the leaves and seeds of velvet beans (*Mucuna pruriens* (L.) var. *utilize*) and in fava beans (*Vicia faba* L.). This compound exhibits allelochemical activity and plays a role in resistance to herbivores. Moreover, L-Dopa is a precursor of other catecholamines, alkaloids or melanin, and, in some cases, might be metabolized to DHCA [38]. Another way to obtain dihydrocaffeic acid is the reduction of the double bond of caffeic acid. Ibdah et al. [39] cloned an NADPH-dependent hydroxycinnamoyl-CoA double bond reductase from *Malus × domestica*. The authors described the possibility of the synthesis of *p*-dihydrocoumarate and dihydroferulate from *p*-coumaroyl-CoA and feruloyl-CoA as substrates, respectively, using the recombinant Malus double bond reductase [39]. Hypothetically, the biosynthesis of DHCA from caffeic acid may be similar to the findings of the above research.

## 4. Metabolism of Dihydrocaffeic Acid by Intestinal and Lactic Acid Bacteria

Phenolic compounds are undoubtedly one of the most valuable substances that we intake with food. Bioavailability, metabolism, and digestion, as well as the pharmacokinetics of food products that are abundant in phenolics, are issues that are gaining the interest of the scientific community, especially due to the progress in analytical chemistry and chromatographic and spectroscopic methods. Dihydrocaffeic acid is a compound that is observed in samples of plasma, urine, or faeces after the consumption of products with a high content of chlorogenic and caffeic acids, which indicates, e.g., the activity of intestinal microbiota on the metabolic fate of these compounds. In recent years, scientists have examined what happens to the phenolic compounds that are ingested along with the following food products: coffee, yerba mate, cocoa, and artichoke, as well as berry, grape, and apple products [40,41,42,43,44,45,46,47,48,49,50].

As one of the most consumed beverages worldwide, coffee is the main source of chlorogenic acid in the human diet. Redeuil et al. [40] conducted research on the coffee metabolites in the plasma of nine people after the ingestion of 400 mL of instant coffee. The authors identified 34 compounds, and they were mainly reduced (dihydrocaffeic acid), methylated (dihydroferulic acid, dimethoxycinnamic acid), and sulfated (dihydroferulic acid 4′-*O*-sulfate and caffeic acid 3′-*O*-sulfate) forms of caffeic acid. Moreover, it was found that the highest concentration of DHCA in plasma was observed 10 h after ingestion and that DHCA 4′-*O*-sulfate was also present in the samples.

In another study, where 13 volunteers consumed one cup of coffee, plasma samples were analyzed by UPLC-MS/MS. According to the findings of the authors, coffee metabolites can be divided into two groups, i.e., the first group of derivatives appeared in plasma in the hour after ingestion and comprised ferulic acid and its sulfated forms, and the second group, which appeared later (about the 4th–6th hour) was linked with the activity of the intestinal microflora. The latter were, for example, dihydroferulic and dihydrocaffeic acid sulfates or feruoylglycine. In the case of DHCA sulfates, these compounds reached the highest plasma concentration, i.e., 0.678 μM 480 min after the consumption of coffee [41].

Furthermore, Scherbl et al. [42] compared the consumption of only instant coffee with coffee plus high-fat or high-carbohydrate meals, and the volunteers ingested 3.1 mg of chlorogenic acid/kg of body weight. The authors revealed that a combination of breakfast and coffee consumption affected a retarded release of coffee metabolites. Among all of the identified compounds, four of them, so-called “colonic metabolites,” namely dihydro-*m*-coumaric acid, dihydroferulic acid, dihydrocaffeic-3′-*O*-sulfate, and dihydroisoferulic acid, had the highest plasma concentrations. In the case of DHCA, the authors observed significant differences in the plasma concentration between the high-carbohydrate meal plus coffee compared to pure coffee consumption [42].

In the studies of de Oliveira et al. [43], Wistar rats were fed with a solution of mate tea or 5-caffeoylquinic acid (5-CQA), and the distribution of the phenolics in tissues (liver, kidney, stomach, small and large intestines, and *biceps femoris* muscle), hepatic and plasmatic kinetics, and urinary excretion were investigated. The presence of dihydrocaffeic acid was confirmed in the kidneys, stomach, and small intestine. Similarly to the above-cited papers, DHCA with other phenolics appeared in the plasma samples, and they were formed after chlorogenic acid metabolism by intestinal bacteria. Dihydrocaffeic acid underwent further metabolism to 3-hydroxyphenylpropionic, 3-hydrobenzoic, and hippuric acids, and some part was also excreted with urine [43].

Cocoa is also a source of phenolics, and cocoa powder consists mainly of epicatechin, (+)-catechin, and procyanidin B2, and became a subject of research by Urpi-Sarda et al. [43]. Both human volunteers’ and rats’ urine samples were analyzed in terms of changes in phenolic profiles before and after cocoa ingestion. In the case of humans, they consumed 40 g of cocoa dissolved in 250 mL of water and samples of their urine were tested before and 24 h after ingestion. Rats were given various diets, with their feed containing ∼4% or 10% (*w*/*w*) of natural cocoa, and were fed for two weeks. In the human and rat urine samples, an increase in DHCA concentration was observed, which resulted in 11, 106, and 267% increases in their concentrations, respectively, depending on the diet used [44].

Next but not least, one food product abundant in caffeic acid and its derivatives is artichoke (*Cynara scolymus* L.). Both artichoke leaves and heads are a source of mono- and di-caffeoylquinic acids and glucosides of luteolin and apigenin. Therefore, the plasma and urine metabolite profiles seemed to be similar to those obtained from coffee or cocoa ingestion. The maximum concentration of dihydrocaffeic and dihydroferulic (DHFA) acids was reached 6–8 h after consumption, which once again confirmed that the simultaneous decrease in caffeic acid concentration and increase in DHCA and DHFA absorption resulted from the hydrolysis of caffeoylquinic acids and the activity of microflora [45,46].

A complete and detailed metabolic fate of dihydrocaffeic acid was investigated by Poquet et al. [51]. The authors carried out metabolism experiments with the use of cell cultures, and elaborated in vitro and ex vivo models for transport studies and liver metabolism, as well as in vivo metabolism studies of 100 µmol DHCA/kg orally administrated to Sprague–Dawley rats. According to the authors’ findings, dihydrocaffeic acid in its free form was absorbed by the stomach, by duodenal or jejunal cells, or more often, when it occurred in an esterified or more complicated form, by the ileum or the colon as a result of intestinal microflora activity. Right after the ingestion, dihydrocaffeic acid was metabolized to its glucuronide, sulphate, or methylated derivatives. The authors identified the following compounds in the plasma samples: 3′- and 4′-*O*-glucuronides and 3′- and 4′-*O*-sulfates of dihydrocaffeic acid, and also dihydroferulic, ferulic, and isoferulic acids. Moreover, the authors claimed that the 3-OH position in the catechol ring was favoured for conjugations or methylation, and, in the intestinal epithelium glucuronidation of DHCA, occurred more often and, oppositely, in the rat liver, sulfation was preferred. Eventually, part of this compound in a free, bound, or metabolized form remained excreted in urine [51].

On the one hand, the metabolism and formation of dihydrocaffeic acid and other phenolic acids in the scientific literature are studied to find out what happens to these compounds after their consumption and what influence the intestinal microflora has on them. On the other hand, it is worth mentioning lactic acid bacteria (LAB) and their activity in food fermentation, with a special emphasis on the formation of functional metabolites. LAB have a broad spectrum of enzymes for the biotransformation of phenolic compounds. One of the most studied enzymes related to phenolic compounds metabolism and, in particular, releasing free phenolic acids, are ferulic acid esterases. These enzymes may be responsible, e.g., for the hydrolysis of chlorogenic acid, methyl ferulate, or methyl vanillate [52,53]. The research is carried out both on single compounds and multi-component food products. In accordance with the findings of Sanchez-Maldonado et al. [54], *Lactobacillus plantarum* and *L. hammesii* were more resistant to the action of 6 hydroxybenzoic and 6 hydroxycinnamic acids in comparison with *E. coli* and *B. subtilis* strains, which is related to the possibility of phenolics metabolism. Biotransformation activity was strain-dependent, but some strains were able to hydrolyze chlorogenic acid, with the subsequent reduction of caffeic acid to dihydrocaffeic acid or decarboxylation to vinylcatechol. Similarly, ferulic acid was reduced to dihydroferulic acid, or phloretic acid appeared when the concentration of *p*-coumaric acid was decreased [54].

Pereira-Caro et al. [55] and Guo et al. [56] investigated the biotransformation of citrus flavanones, namely hesperetin-7-*O*-rutinoside and naringenin-7-*O*-rutinoside, by different strains of lactic acid bacteria. Both studies confirmed the possibility of bacterial metabolism of these flavanones. Some strains exhibited rhamnosidase and glucosidase activity; hence, flavanones were converted firstly to their aglycones, and then ring fission occurred [55,56]. In the case of hesperetin-7-*O*-rutinoside, the following compounds were formed due to the activity of *Bifidobacterium longum* R0175 and *L. rhamnosus* subsp. *Rhamnosus* NCTC 10302: 3,4-dihydroxyphenylpropionic (DHCA), 3-hydroxyphenylpropionic, and phenylpropionic acids. For the second flavanone, 4-hydroxyphenylpropionic and phenylpropionic acids were observed [55].

The formation of dihydrocaffeic acid due to the fermentation processes of lactic acid bacteria was also observed in varied sourdoughs (wheat or sorghum), cherry juices, and fermented milk enriched with an extract from the leaves of *Cudrania tricuspidata* [57,58,59,60,61]. Among three tested strains of lactic acid bacteria that were used for the fermentation of wheat flour, only one, namely *L. amylovorus* DSM19280, was able to produce dihydrocaffeic acid, and its concentration was determined to be 1.2 ± 0.1 mg/kg of sourdough [57]. Oppositely, when *Furfurilactobacillus milii* FUA3583 was applied for sorghum grains fermentation, the results revealed that the total concentration of free phenolic acids increased and that DHCA had the highest concentration. Furthermore, the authors disrupted several genes, coding the reductase, decarboxylase, and esterase of *F. milii*, and when the hydroxycinnamic acid reductase Par1 was deleted, the Δpar1Δpar2 reductase mutant produced very low levels of DHCA [58].

The cherry juice, consisting mainly of caffeic, *p*-coumaric, and protocatechuic acids, was subjected to a fermentation process at 30 and 37 °C by different LAB strains. Phenolic acids, during the fermentations, were metabolized to their derivatives, where caffeic acid was converted to DHCA. Only strains of *L. plantarum* had the appropriate enzymes to increase the level of dihydrocaffeic acid in the fermented cherry juice. The 285 strain of *L. plantarum* was able to metabolize all of the phenolics from the cherry juice, after 48 h, to phenyllactic acid, *p*-hydroxyphenyllactic acid, and dihydrocaffeic acid. On the other hand, the POM1 strain formed the highest amount of DHCA, and its concentration reached 0.609 µg/mL [59]. Similarly, in the studies of Filannino et al. [60], cherry juice and, additionally, broccoli puree, were fermented with *Lactobacillus* spp. Once again, dihydrocaffeic acid was found to be a major caffeic acid derivative during the fermentation of cherry juice. In the case of broccoli pure, only the strains *L. fermentum* FUA3165 and *L. reuteri* FUA3168 produced slight amounts of DHCA from chlorogenic and caffeic acids [60].

A different study, or rather, a different fermented product, was presented by Oh et al. [61]. The authors applied four different strains of *L. gasseri*, a probiotic isolate of human origin, for the fermentation of milk that was enriched with the leaf extract of *Cudrania tricuspidata*. This herb is used in Asian folk medicine and is abundant in chlorogenic, neochlorogenic, and caffeic acids, as well as in flavonoids such as rutin and glycosides of quercetin and kaempferol. Phenolic acids were predominantly metabolized by the used LAB strains, with the simultaneous formation of DHCA as a major derivative formed during fermentation. Caffeic acid was the compound whose concentrations decreased the most, and in the case of dihydrocaffeic acid, after 48 h, its concentration ranged from 82 to 108 μg/g dry matter depending on the *L. gasseri* strain [61].

As can be seen in the literature, lactic acid bacteria have the appropriate enzymatic features to affect the polyphenol profile of many food products. Food that is rich in chlorogenic or caffeic acids, as well as their derivatives, in many cases confirmed by the above examples, fermented by lactic acid bacteria, led to the formation of other phenolic compounds than in the initial product, where dihydrocaffeic acid was often one of the main representatives.

## 5. Recent Advances in the Enzymatic Synthesis of Dihydrocaffeic Acid Derivatives

**Figure 4:**
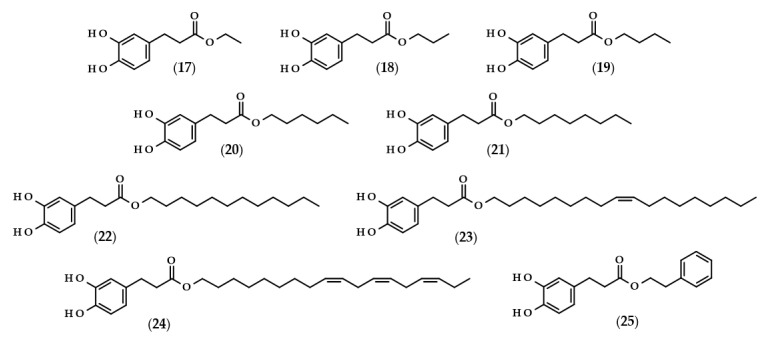
Chemical structures of dihydrocaffeic acid esters obtained with the use of enzymatic processes. Chemical structures of: ethyl dihydrocaffeate (**17**), propyl dihydrocaffeate (**18**), butyl dihydrocaffeate (**19**), hexyl dihydrocaffeate (**20**), octyl dihydrocaffeate (**21**), dodecyl dihydrocaffeate (**22**), oleyl dihydrocaffeate (**23**), linolenyl dihydrocaffeate (**24**), and phenethyl dihydrocaffeate (**25**).

For many phenolic compounds, due to their hydrophilic character, the problem is their application in organic solvents or high-lipid food matrices. One of the solutions to this issue may be the use of enzymatic processes, including the use of lipases for the lipophilization of phenolic compounds, i.e., esterification of the carboxyl or hydroxyl group with long-chain alcohol or carboxylic acid, respectively, with the enzyme as a biocatalyst. The main goal of lipophilization should be to increase compounds’ solubility in organics, but simultaneously, very frequently, their biological activity changes. Moreover, enzymatic reactions in comparison to conventional chemical processes are more environmentally friendly, which is due to less energy consumption and waste generation [62,63]. Examples of such modifications relating to dihydrocaffeic acid are shown below, and the chemical structures of the described derivatives are presented in Figure 4.

To the best of the author’s knowledge, the first manuscript relating to the enzymatic modification of dihydrocaffeic acid was published in 1997 by Guyot et al. [64]. The authors applied various phenolic acids to direct esterification with linear alcohols, with *Candida antarctica* as a biocatalyst. According to their findings, the esterification yields ranged from 3 to 98% and were dependent on the substituents in the phenolic ring and the presence of double bond in the propenoic chain, as well as the length of the alcohol used. In the case of DHCA, the yields were always higher than 65% when the following alcohols were compared: butanol (**19**), octanol (**21**), dodecanol (**22**), and 9-octadecen-1-ol (**23**) [64]. A similar esterification yield, i.e., 67%, was also obtained by Zieniuk et al. [1] when DHCA was esterified with butanol with the same biocatalyst. Weitkamp et al. [65] investigated the transesterification of several methyl or ethyl esters of phenolic acids with *cis*-9-octadecen-1-ol. The reaction yield of oleyl dihydrocaffeate (**21**) was significantly higher in comparison with its saturated derivative, and was also higher than for oleyl ferulate and oleyl sinapate [65]. This confirmed that the presence of a double bond in the side chain of phenolic acids negatively affects the enzymatic activity and the final yield of the reaction.

In a similar way, the lipophilization of dihydrocaffeic acid was also carried out by Sørensen et al. [66] and Sabally et al. [67]. In the first paper, Novozyme 435 (*C. antarctica* lipase B, CALB) was applied for the synthesis of octyl and oleyl esters of DHCA (**21** and **23**) [66]. A detailed analysis of the enzymatic esterification conditions was studied in the second paper. The authors evaluated the composition of solvent mixtures and phenolic acid-to-alcohol ratios on the course of DHCA lipophilization with linolenyl alcohol. Dihydrocaffeic acid is slightly soluble in nonpolar organic solvents, but CALB acts better in nonpolar environments; hence, the authors proved that the solvent mixtures of hexane and butan-2-one of 75:25 (*v*/*v*) give a higher esterification yield (76%) in comparison with the more polar mixture (65:35 (*v*/*v*) and 58% of conversion). Moreover, it was revealed that the higher the concentration of linolenyl alcohol, the higher the conversion that was achieved. Therefore, when the ratio of 1:8 was applied, the authors were able to obtain a 99% conversion of linolenyl dihydrocaffeate (**24**) [67].

Scientists are looking for improvements that will increase their conversion rate when dihydrocaffeic acid is lipophilized using enzymes and hydrophobic alcohols. It seems to be important to apply the appropriate solvent that can dissolve the reactants, that is, hydrophilic phenolic acid and lipophilic alcohol, and that, at the same time, keeps the enzyme active. As a consequence, ionic liquids have been tested as solvents in the lipase-catalyzed processes. Trioctylmethylammonium trifluoroacetate (tOMA.TFA) was an example of the ionic liquid used for the synthesis of octyl dihydrocaffeate (**21**). It was shown that the temperature of 70 °C and the ratio of 12:1 (*v*/*v*) octan-1-ol/tOMA.TFA, corresponding to a octan-1-ol/DHCA ratio of 38:1 (mol/mol), were necessary to achieve the maximum conversion of DHCA to its octyl ester [68]. Gholivand et al. [69], besides the use of ionic liquids, supplemented their research with the optimization of lipase-catalyzed esterification of dihydrocaffeic acid with hexanol by response surface methodology (RSM) and, in addition, a central composite design (CCD) was employed. According to the obtained results, the highest possible esterification yield of hexyl dihydrocaffeate (**20**), that is 84.4%, was achieved within the following conditions: a DHCA/hexanol ratio of 1:2, 41.6% of the enzyme, 77.5 h the process at 39.4 °C, and, additionally, with (1-butyl-3-methylimidazoliumbis (trifluoromethylsulfonyl) imide) as a solvent [69].

A different approach to the synthesis of dihydrocaffeic acid derivatives was presented by Bozzini et al. [70] and Botta et al. [71]. The syntheses of methyl (**2**), ethyl (**17**), propyl (**18**), and butyl esters (**19**) of DHCA were two-stage chemo-enzymatic processes, that is, 3-(4-hydroxyphenyl)propanoic acid was firstly esterified to its corresponding esters with the use of trimethylchlorosilane (TMCS) at 25 °C. Subsequently, the resulting esters were oxidized to their catechol derivatives and, for that purpose, tyrosinase from the mushroom *Agaricus bisporus* was used [70,71].

Among the enzymatic methods of obtaining esters of phenolic acids, a place should also be devoted to a chlorogenate hydrolase (EC 3.1.1.42). Oppositely to the lipase-catalyzed reactions, Kishimoto et al. [72] demonstrated the possibility of using this enzyme in an aqueous environment, consisting of citrate buffer for the synthesis of phenethyl dihydrocaffeate (**25**).

**Figure 5:**
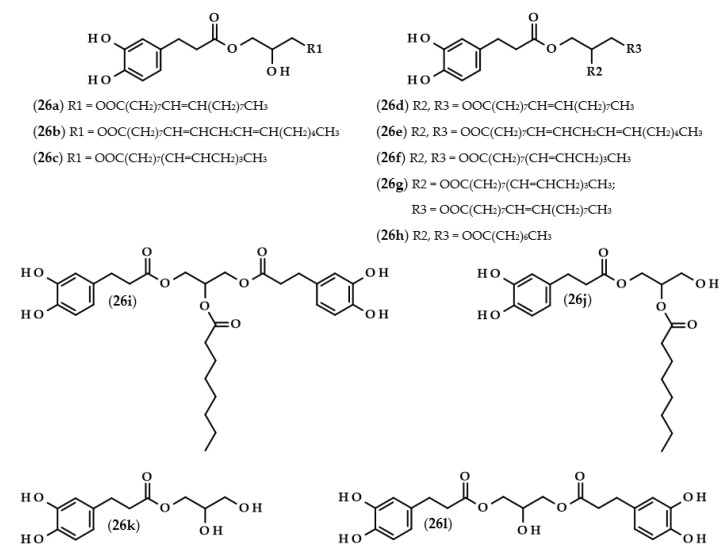
Chemical structures of phenolipids with dihydrocaffeic moiety/moieties. Chemical structures of: 1-dihydrocaffeoyl-3-oleoylglycerol (**26a**), 1-dihydrocaffeoyl-3-linoleoylglycerol (**26b**), 1-dihydrocaffeoyl-3-linolenoylglycerol (**26c**), 1-dihydrocaffeoyl-2,3-dioleoylglycerol (**26d**), 1-dihydrocaffeoyl-2,3-dilinoleoylglycerol (**26e**), 1-dihydrocaffeoyl-2,3-dilinolenoylglycerol (**26f**), 1-dihydrocaffeoyl-2-linolenoyl-3-oleoylglycerol (**26g**), 1-dihydrocaffeoyl-2,3-dicapryloylglycerol (**26h**), 1,3-bis(dihydrocaffeoyl)-2-capryloylglycerol (**26i**), 1-dihydrocaffeoyl-2-capryloylglycerol (**26j**), dihydrocaffeoylglycerol (**26k**), and 1,3-bis(dihydrocaffeoyl)glycerol (**26l**).

Phenolipids or lipophenols (**26**, Figure 5) are a group of phenolic compounds which can be modified throughout lipophilization. The main goal of this approach is to obtain hydrophobic compounds which possess antioxidant properties [73]. In describing the next few papers, this term will refer to triacylglycerols, which, as a result of the modification, acquired a phenolic acid moiety instead of the fatty acid residue.

Several research groups have investigated the lipase-catalyzed transesterification of DHCA with triacylglycerols [74,75,76]. In the research of Sabally et al. [74], it was proven that the number of the obtained products in the reaction between dihydrocaffeic acid and flaxseed oil, as well as their yields, were dependent on the molar ratio of the reactants. When the authors used equimolar concentrations of the substrates, only phenolic monoacylglycerols were synthesized. By increasing the concentration of the flaxseed oil, phenolic mono and diacylglycerols were obtained, and the yield of phenolic diacylglycerols increased with an increasing reactant ratio. Furthermore, the authors confirmed the synthesis of the following phenolipids: monooleyl dihydrocaffeate (**26a**), monolinolenyl dihydrocaffeate (**26c**), dioleyl dihydrocaffeate (**26d**), dilinoleyl dihydrocaffeate (**26e**), dilinolenyl dihydrocaffeate (**26f**), and oleyl linolenyl dihydrocaffeate (**26g**) [74]. The same authors continued research on this subject and, in the biosynthesis of phenolipids, trilinolein and trilinolenin were used. Application of the first triacylglycerol led to the maximum conversion of 66% after five days with the ratio of 1:2 (DHCA:trilinolein) and both mono- and di-linoleyl dihydrocaffeates (**26b** and **26e**) were obtained. In the case of the second lipid, a similar yield (62%) of the reaction was achieved only after 12 days. Likewise, mono- and di-linolenyl dihydrocaffeates (**26c** and **26f**) were confirmed using LC-MS [75].

A completely distinct way of obtaining phenolated acylglycerols was elaborated and presented by Yang et al. [76]. The biosynthesis of these compounds was reported as a two-step process. Firstly, the enzymatic synthesis of octyl dihydrocaffeate (**21**) from DHCA and octanol occurred. Subsequently, an enzymatic interesterification was the second step, in which the triacylglycerol reacts with octyl dihydrocaffeate. The authors compared three different enzymes, and investigated the usefulness of octyl ester instead of phenolic acid as well as the presence or the absence of the solvent. It was shown that the highest conversion (72.6%) was achieved when octyl dihydrocaffeate and tricaprilin were used in a ratio of 1:2, and when Novozym 435 was applied in the solvent-free reaction. The obtained products were 1-dihydrocaffeoyl-2-capryloylglycerol (**26j**), 1,3-bis(dihydrocaffeoyl)-2-capryloylglycerol (**26i**), 1-dihydrocaffeoyl-2,3-dicapryloylglycerol (**26h**), dihydrocaffeoylglycerol (**26k**), and 1,3-bis(dihydrocaffeoyl)glycerol (**26l**) [76].

**Figure 6:**
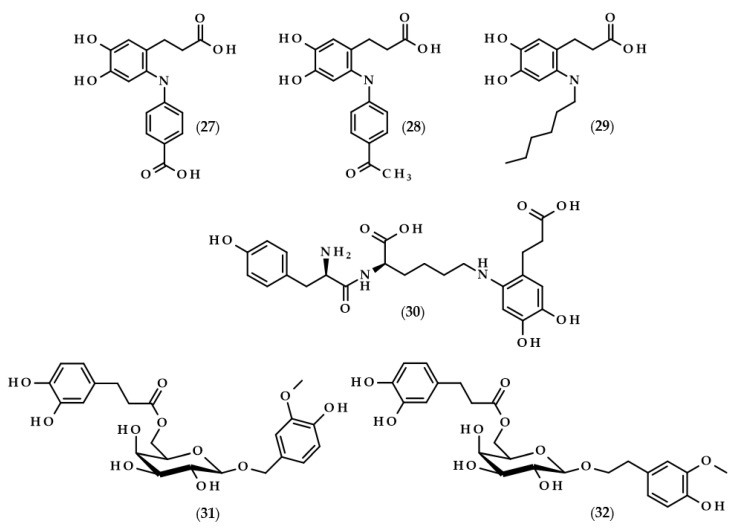
Chemical structures of other dihydrocaffeic acid derivatives obtained in the enzymatic processes, i.e., 3-[6-(4-carboxyphenyl)amino-3,4-dihydroxyphenyl]propanoic acid (**27**), 3-[6-(4-acetophenyl)amino-3,4-dihydroxyphenyl]propanoic acid (**28**), 3-(6-hexylamino-3,4-dihydroxyphenyl)propanoic acid (**29**), 3-[6-tyrosyllysine-3,4-dihydroxyphenyl]propanoic acid (**30**), 2-(4-hydroxy-3-methoxyphenyl)methyl-6-*O*-dihydrocaffeoyl-β-D-galactopyranoside (**31**), and 2-(4-hydroxy-3-methoxyphenyl)ethyl-6-*O*-dihydrocaffeoyl-β-D-galactopyranoside (**32**).

Another example of enzymes used to modify dihydrocaffeic acid are laccases. These enzymes (EC 1.10.3.2) are copper-containing oxidases that are present in plants, fungi, and other microorganisms. Laccases catalyze the oxidation of a broad spectrum of phenolic compounds to phenoxyl radicals with the simultaneous four-electron reduction of molecular oxygen to water. They are also called lignin-modifying enzymes and play a crucial role in the biodegradation of lignin. The application of fungal laccases was investigated by Pilz et al. [77] and Chaurasia et al. [78]. The cross-coupling reaction of dihydrocaffeic acid and 4-aminobenzoic acid was catalyzed by laccase from *Pycnoporus cinnabarinus* DSM 15225, which was white rot fungus isolated from an oak tree. The reaction, carried out in a stirred tank reactor, allowed for the conversion of 81.1% of DHCA after 120 min, and one cross-coupling product, i.e., 3-[6-(4-carboxyphenyl)amino-3,4-dihydroxyphenyl] propanoic acid (**27**, Figure 6), was synthesized [77].

The research was continued by Chaurasia et al. [78]. This time, the fungus laccase was used and three reactions were carried out. The first was identical to the above paper and the next two, were carried out with 4-aminoacetophenone and 1-hexylamine, respectively. The reactions proceeded in sodium acetate buffer (pH 5.0) at room temperature with purified laccase from *Pleurotus sajor caju* MTCC-141. After roughly 4 h, yields of 90, 86, and 75% were achieved for the corresponding cross-coupling compounds 3-[6-(4-carboxyphenyl)amino-3,4-dihydroxyphenyl]propanoic acid (**27**), 3-[6-(4-acetophenyl)amino-3,4-dihydroxyphenyl]propanoic acid (**28**), and 3-(6-hexylamino-3,4-dihydroxyphenyl)propanoic acid (**29**) [78]. 

Mikolasch et al. [79] focused on the synthesis of potential biomaterials based on tyrosyllysine (Tyr-Lys) and dihydroxylated phenolic acids. In order to achieve their goal, the laccase-catalyzed cross-linking reactions were applied, and the fungus *P. cinnabarinus* SBUG-M 1044 was the source of this enzyme. The authors obtained the dimer from the reaction of dihydrocaffeic acid (as a substrate of the laccase) and tyrosyllysine, namely 3-[6-tyrosyllysine-3,4-dihydroxyphenyl]propanoic acid (**30**), and moreover, four different trimers were also detected by MS and NMR techniques. The key point of their work was also accomplished, that is, the successful synthesis of a cross-linked polymer between oligopeptide [Tyr-Lys]_10_ and DHCA [79].

The application of enzymes to modify phenolic compounds does not always have to relate to the lipophilization of these compounds and increasing their hydrophobic character. Lopez-Munguia et al. [80] were pioneers in the enzymatic synthesis of phenylpropanoid glycoside analogues. In contrast to conventional chemical syntheses, their approach allowed for obtaining considerable yields (40–60%) without the protection of the functional groups and with a two-step, completely enzymatic process. The first step required the synthesis of vanillyl and homovanillyl alcohol galactosides by *Kluyveromyces lactis* β-galactosidase transgalactosylation reactions. Galactosides were produced in saturated lactose solutions in phosphate buffer (pH 6.5) at 35 °C. Interestingly, the use of high concentrations of lactose caused the favoring of transgalactosylation over hydrolysis, thus resulting in 30–35% yields. In the second step, the authors esterified galactosides with saturated and unsaturated phenolic acids, namely (dihydro)ferulic and (dihydro)caffeic acids. During 24 h reactions, CALB, as a biocatalyst, was successfully applied in the formation of 2-(4-hydroxy-3-methoxyphenyl)methyl-6-*O*-dihydrocaffeoyl-β-D-galactopyranoside (**31**) and 2-(4-hydroxy-3-methoxyphenyl)ethyl-6-*O*-dihydrocaffeoyl-β-D-galactopyranoside (**32**), with yields of approximately 50%. Phenylpropanoid glycoside analogues were also obtained when ferulic and dihydroferulic acids were used, but only traces of the investigated compound were found when caffeic acid was chosen, which is related to the lower electrophilicity of the carboxylic functional groups of the unsaturated phenolic acids [80].

## 6. Biological Activity of Dihydrocaffeic Acid and Its Derivatives

**Figure 7:**
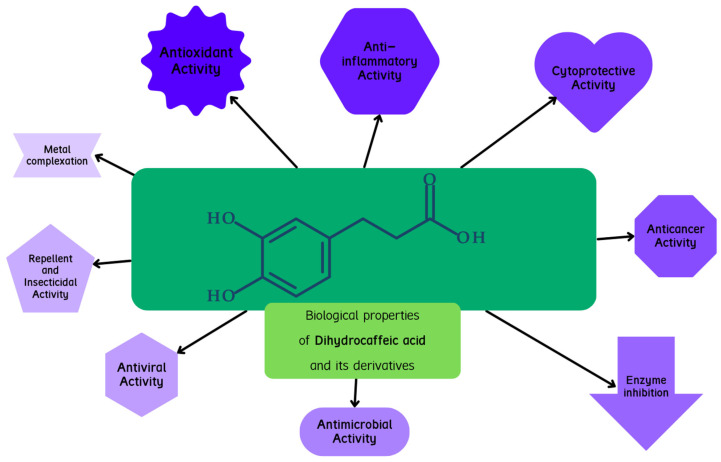
Graphical presentation of biological activities attributed to dihydrocaffeic acid and its derivatives.

A graphical presentation of the activity of dihydrocaffeic acid and its derivatives is shown in Figure 7. The biological activities, including the antioxidant, cytoprotective, anticancer, and antimicrobial activities of these compounds, are described below.

### 6.1. Antioxidant Activity

Probably the most frequently undertaken research in the case of phenolic compounds is the study of their antioxidant activity. The most common antioxidant assays are based on simple chemical reactions between an antioxidant and model free radicals such as 2,2-diphenyl-1-picrylhydrazyl radical (DPPH^•^) or the radical cation of 2,2′-Azino-bis(3-ethylbenzothiazoline-6-sulfonic acid) diammonium salt (ABTS^+•^), or on metal ions, as in FRAP (Ferric Reducing Antioxidant Power) and CUPRAC (CUPric Reducing Antioxidant Capacity) methods. As well, in the case of dihydrocaffeic acid and its derivatives, these tests were successfully used [1,5,25,26,81,82,83,84].

Twelve isolated and identified compounds from *G. bicolor* stems and leaves were compared by means of DPPH and ABTS radical scavenging assays. Among the tested compounds, dihydrocaffeic acid and its methyl ester (**2**) were present. Scavenging effects were demonstrated as IC_50_ values, that is the concentration needed to achieve a 50% reduction in the radicals, where the ester was less active and values of 1.22 and 1.38 mM for the DPPH and ABTS methods were obtained, respectively. In the case of phenolic acid, the IC_50_ values were two–three times lower (0.44 and 0.49 mM), which indicated a much higher radical scavenging activity [5]. In the research of Zieniuk et al. [1], butyl dihydrocaffeate (**19**) had lower activity than DHCA when the DPPH assay was employed, but, oppositely, in the CUPRAC test, butyl ester exhibited the highest activity also in comparison with L-ascorbic acid or butylated hydroxytoluene, and similar activities were observed for gallic and caffeic acids [1]. Silva et al. [81] evaluated the antioxidant activity of DHCA and methyl (**2**), ethyl (**17**), and propyl (**18**) esters, as well as their corresponding unsaturated derivatives. According to their findings, dihydrocaffeic acid proved to be the most active compound in the DPPH assay, with antiradical activity higher than that of tocopherol. The formation of esters led to a dramatic decrease in antioxidant activities, but the chain length had no effect on the exact activity [81].

Due to the use of different protocols for the determination of antioxidant activity, it happens that the results presented in different publications are contradictory or difficult to compare. Therefore, Nenadis and Tsimidou [82] examined the duration of the test, the molar ratio of antioxidant to DPPH^•^, and the solvent (ethanol, acetonitrile, and *tert*-butanol). The authors used several phenolic acids, phenolic alcohols (tyrosol and hydroxytyrosol), oleuropein, and other known antioxidants to evaluate their concept. It was proven that the solvent choice is critical to assess antioxidant activity, and the following conditions: 20 min reaction period and molar ratio causing a 60–80% radical scavenging activity, were suggested. In the case of dihydrocaffeic acid, its behaviour was dependent on the used solvent, where its highest activity among all of the compounds was observed in ethanol, then in *tert*-butanol, which had a comparable activity with caffeic acid, and, finally, DHCA, which was less active than caffeic and rosmarinic acids when acetonitrile was used as a solvent [82].

**Figure 8:**
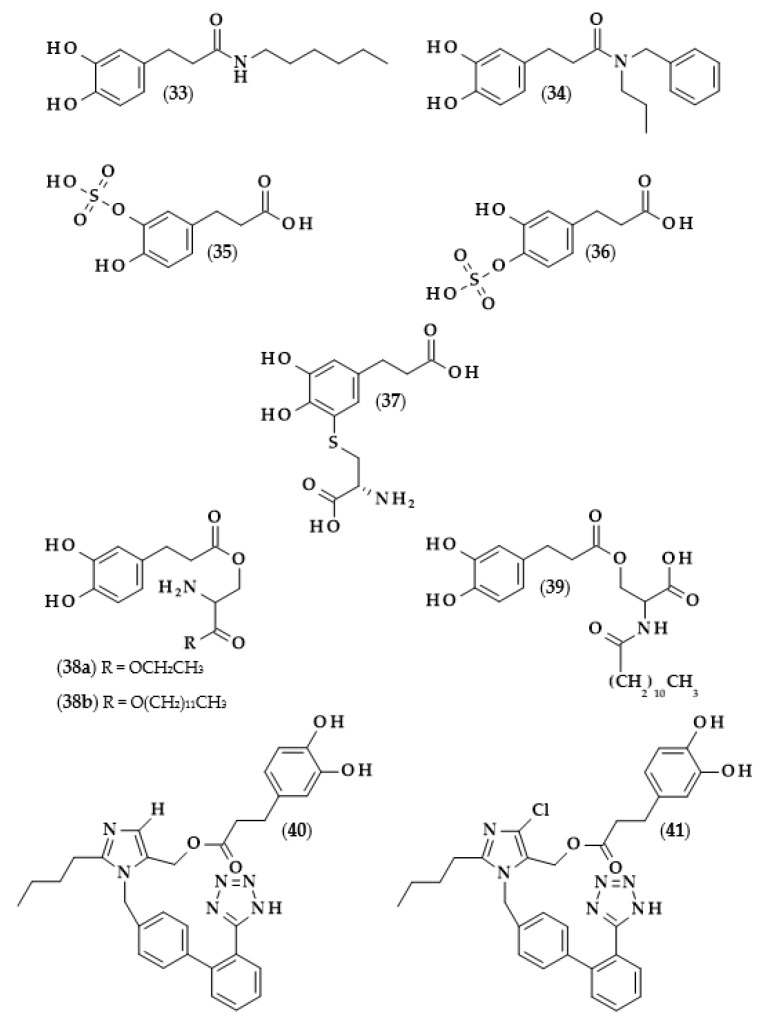
Chemical structures of: 3-(3,4-dihydroxyphenyl)-*N*-hexylpropanamide (**33**), *N*-benzyl-3-(3,4-dihydroxyphenyl)-*N*-propylpropanamide (**34**), dihydrocaffeic acid-3-*O*-sulfate (**35**), dihydrocaffeic acid-4-*O*-sulfate (**36**), 5′-cysteinyl dihydrocaffeic acid (**37**) *O*-(3,4-dihydroxyphenyl-3-propanoyl)-L-serine ethyl ester (**38a**), *O*-(3,4-dihydroxyphenyl-3-propanoyl)-L-serine lauryl ester (**38b**), *O*-(3,4-dihydroxyphenyl-3-propanoyl)-*N*-lauroyl-L-serine (**39**), dihydrocaffeic acid 2-butyl-3-[2’-(*2H*-tetrazol- 5-yl)-biphenyl-4-ylmethyl]-*3H*-imidazol-4-yl methyl ester (**40**), and dihydrocaffeic acid 2-butyl-5-chloro-3-[2′-(*2H*-tetrazol-5-yl)-biphenyl-4-ylmethyl]-*3H*-imidazol-4-yl methyl ester (**41**).

Hexylesters and hexylamides of caffeic and ferulic acids, and their saturated analogues, were synthesized by Roleira et al. [83]. Due to the presence of a catechol ring, hexylamide of dihydrocaffeic acid (3-(3,4-dihydroxyphenyl)-*N*-hexylpropanamide, (**33**), Figure 8), hexyl dihydrocaffeate (**20**), and derivatives of caffeic acid were the most active compounds. Considering the results achieved with the tests with DPPH and ABTS, as in the above-cited articles, lipophilic derivatives had much lower activities than the parent compounds. In both tests, it was observed that the activity of DHCA was higher than that of caffeic acid. The Trolox equivalent antioxidant capacities (TEAC) for dihydrocaffeic acid were: 2.05 in the DPPH assay, and 1.16 and 1.41 in the ABTS method, after 5 and 20 min, respectively. The aim of the authors was the synthesis of compounds that would have antioxidant activity and, at the same time, be able to cross the blood–brain barrier. Hence, the use of spectrophotometric tests in rather hydrophilic environments was not suitable; therefore, all of the compounds were applied in the lipoperoxidation assay. It was revealed that caffeic acid ester and amide were more effective than saturated analogues. Considering only hydrogenated compounds, hexyl dihydrocaffeate with a TEAC value of 3.49 was the most active chemical, followed by hexylamide derivative (2.08), and the parent acid had a TEAC of 1.75 [83].

Other derivatives of the described phenolic acid which were subjected to antioxidant activity measurements were dihydrocaffeic acid-3-*O*-sulfate (**35**) and dihydrocaffeic acid-4-*O*-sulfate (**36**) [84], i.e., the compounds that are formed during the metabolism of chlorogenic, caffeic, and dihydrocaffeic acids [51]. In the case of dihydrocaffeic acid, as well as other phenolic acids used in the study, it was observed that sulfation of the hydroxyl group in the aromatic ring caused a significant reduction in antioxidant activity. Comparing DHCA to its monosulfate derivative (mixture of both sulfates at an unknown ratio), the authors obtained the following results: (a) in the CUPRAC method, 0.49 mM of Trolox equivalents (TE) for DHCA, and no activity for monosulfates; (b) using Folin–Ciocalteu assay, 72.07 to 2.19 ppm of gallic acid equivalents; (c) in the DPPH radical test, 0.563 mM of TE (39.96% of scavenging effect), which was approximately 10 times higher than the values for monosulfates (0.051 mM and 3.61%) [84].

Explanations of the differences in the antioxidant activity of phenolic compounds in connection with the presence or absence of a double bond in the carbon chain, as well as the number and arrangement of substituents in the aromatic ring, were undertaken several times using both experimental and computational (using the density functional theory (DFT)) approaches for Structure-Activity Relationships studies [85,86,87,88].

The authors took into account the role of the carbon side chains of caffeic and dihydrocaffeic acids. Several methods were applied for the comparison, but the results from the free radicals tests that they conducted were the most important for the attempt to explain the chosen subject of research. The unsaturated bond found in the carbon chain of caffeic acid may participate in the stabilization of the phenoxyl radicals by resonance. Due to the presence of the catechol ring in both compounds, the observed results were comparable and disputable, and this group probably masked the influence of other structural differences in phenolic acids [85]. DFT studies conducted by Bakalbassis et al. [86] provided a good molecular descriptor, i.e., the heat of formation (ΔHOF). Caffeic acid exhibited higher antioxidant activity and showed a lower value of ΔHOF and a higher possibility of delocalization than dihydrocaffeic acid. The authors explained that the higher the ΔHOF value, the more difficult it is to break the O-H bond in the phenolic ring, and, moreover, small amounts of localized spin decrease the chance to initiate a radical chain reaction [86]. In the subsequent years, in other studies conducted on this subject, the results were highly discussed. Siquet et al. [87] performed a structure-antioxidant activity relationship study of di- and tri-hydroxyphenolic acids. It has been shown that the number of hydroxyl groups in the phenyl ring and the alkyl spacer type between the carboxylic functionality and the phenyl ring play a key role in its antioxidant activity. The obtained TEAC values in ABTS and DPPH assays were higher for DHCA than caffeic acid, and to a similar but lesser extent, observation has been reported for 3-(3,4,5-trihydroxyphenyl)propanoic acid and 3-(3,4,5-trihydroxyphenyl)propenoic acids. In the lipoperoxidation test, there were no differences between saturated and unsaturated phenolic acids [87]. The findings of the computational studies of Leon-Carmona et al. [88] revealed that the antioxidant activity was also dependent on the environment of the conducted analyses. Dihydrocaffeic acid was found to be the most reactive compound in the non-polar environments and acidic aqueous solution (pH ≤ 4.5) compared to caffeic, ferulic, and *p*-coumaric acids. Interestingly, when a physiological pH solution was used, ferulic and caffeic acids were more efficient than DHCA. The electron-donating character of the carbon side chain of DHCA and the mechanism of hydrogen atom transfer in the antioxidant action influenced its higher activity in non-polar and acidic aqueous solutions [88].

In the conducted research on the applications of dihydrocaffeic acid and its derivatives, the possibility of their use as antioxidants in oils and emulsions was also considered. The application of DHCA in lard stored at 60 °C caused enhanced oxidative stability in comparison with caffeic acid. In the same study, Moon and Terao [89] also evaluated the inhibition of the copper ion-induced oxidation of human low-density lipoprotein (LDL) with these phenolics, and both compounds exhibited antioxidant activity, but the induction period obtained with DHCA was shorter than that with caffeic acid [89]. The antioxidant activity of DHCA was also shown in plasma and in erythrocytes, where DHCA found in human erythrocytes enhanced the reduction of ferricyanide, thus protecting erythrocytes from oxidative stress [90].

Octyl (**21**) and oleyl (**23**) dihydrocaffeates were used as antioxidants in fish oil-buffer and fish oil-enriched milk emulsions to prevent oxidative changes during storage [66,91]. It was found that octyl ester was more efficient than oleyl ester in emulsions and exhibited a significantly higher effect than DHCA. The authors explained the obtained results with two phenomena. The first one, the so-called “polar paradox,” suggests that better solubility in a given environment leads to higher antioxidant activity; thus, esters were better than parent acid. The second hypothesis—the “cut-off effect”—indicates that esters with long alkyl chains, instead of being at the interface or in the organic phase, form micelles in the aqueous phase, which reduces the antioxidant activity of these lipophilized derivatives [66,91].

Phenolics with catechol rings are well-known for their high antioxidant activity. Unusually, some authors described their pro-oxidant properties, especially in the oxidation of oxymyoglobin (MbO_2_), which is responsible for the bright red color of meat, and, due to its instability, is oxidized to a brownish metmyoglobin. In the study of Masuda et al. [92], several compounds with catechol moiety showed the promotion of myoglobin oxidation. A detailed investigation of the influence of DHCA on the stability of MbO_2_ indicated that the addition of amino acids and especially L-cysteine inhibited the effect of phenolic acids. The authors, continuing their research, synthesized 5′-cysteinyl dihydrocaffeic acid (**37**) using tyrosinase, which might be also formed during oxidation processes, thus probably contributing to the inhibition of MbO_2_ oxidation; therefore, experimentally confirming the inhibitory effect of this compound [92].

Another amide derivative of DHCA, namely *N*-benzyl-3-(3,4-dihydroxyphenyl)-*N*-propylpropanamide (**34**), in combination with phosphatidylcholine, was successfully applied in frying experiments on canola oil [93]. The application of this combination of compounds led to the higher thermo-oxidative stability of canola oil, which was acknowledged by a lesser amount of polar compounds formation (oxidized and oligomerized lipids or free fatty acids and mono- or di-acylglycerols), lower Anisidine Value (a measure of the unsaturated aldehydes formed), and higher concentrations of polyunsaturated fatty acids and tocopherols. It is also worth noting that the amide derivative and phosphatidylcholine acted synergistically through the decomposition of hydroperoxides formed after the reaction of the phenolic derivative with lipid peroxy radicals. Moreover, compound (**34**) was also able to remove the oxidation products, i.e., aldehydes, through the reaction of the carbonyl group with the amine nitrogen [93].

The effects of the combination of serine ethyl ester, serine lauryl ester, and lauroyl serine with several well-known antioxidants, that is Trolox and phenolic acids, and their use as antioxidants in various model systems, were investigated by Hunneche et al. [94]. Among the synthesized compounds, the following DHCA derivatives were obtained: *O*-(3,4-dihydroxyphenyl-3-propanoyl)-L-serine ethyl ester (**38a**), *O*-(3,4-dihydroxyphenyl-3-propanoyl)-L-serine lauryl ester (**38b**), and *O*-(3,4-dihydroxyphenyl-3-propanoyl)-*N*-lauroyl-L-serine (**39**). It was again confirmed that the lipophilization of dihydrocaffeic acid led to improved oxidative stability in heterogeneous systems, and that an important factor on which this activity depends was the octanol/water partition coefficient [94].

Finally, due to the fact that the use of simple spectrophotometric tests on single chemical reactions does not necessarily reflect the actual activity of these compounds in more complex models or in a living organism, the antioxidant activity of the described phenolic acid and its derivatives was also tested in cell cultures [95,96,97].

Huang et al. [95] found that dihydrocaffeic acid was taken up by the human endothelial EA.hy926 cells and acted as an intracellular antioxidant. The oxidant stress in cells was induced by 2,2′-Azobis(2-methylpropionamidine) dihydrochloride (AAPH), a water-soluble free radical initiator, and the antioxidant effects were observed both on DHCA and tocopherol, which were evaluated by a decreased oxidation of *cis*-parinaric acid. In the second experiment, menadione was used for oxidant stress induction, and DHCA also had the ability to decrease intracellular stress and inhibited the oxidation of dihydrofluorescein [95].

Interestingly, yerba mate phenolic extract and DHCA, but not dihydroferulic acid, turned out to be compounds that protect human hepatoma HepG2 cells from the oxidative damage induced by *tert*-butylhydroperoxide. The concentrations of this phenolic acid, i.e., 0.2, 1, and 10 μM, did not show any cytotoxic effect on this cell line, and furthermore, decreased the generation of reactive oxygen species (ROS) and prevented macromolecular damage. The application of DHCA in concentrations of 1 and 10 μM also led to an increase in the concentration of GSH in HepG2 cells, that is, reduced glutathione, a tripeptide associated with the reduction of ROS [96].

Losartan is a drug used to treat high blood pressure (hypertension) and is responsible for inhibiting the action of angiotensin II. Garcia et al. [97] have undertaken the syntheses of losartan derivatives by combining this medicine with various antioxidant moieties and, among them, dihydrocaffeic acid was also used. Dihydrocaffeic acid 2-butyl-3-[2′-(*2H*-tetrazol-5-yl)-biphenyl-4-ylmethyl]-*3H*-imidazol-4-yl methyl ester (**40**) and dihydrocaffeic acid 2-butyl-5-chloro-3-[2′-(*2H*-tetrazol-5-yl)-biphenyl-4-ylmethyl]-*3H*-imidazol-4-yl methyl ester (**41**) were obtained and exhibited the highest antioxidant activity in the ABTS assay, and were also able to inhibit angiotensin II bindings. Subsequently, in vivo experiments with Wistar rats treated with *Nω*-nitro-L-arginine methyl ester hydrochloride (L-NAME), i.e., rats in which hypertension was induced, were conducted. The generation of ROS is partially involved in vascular damage and cardiovascular diseases; hence, the combination of losartan and dihydrocaffeic acid seemed reasonable. The application of (**40**) and (**41**) decreased and normalized systolic blood pressure in rats. Both compounds normalized all of the changes induced in vascular walls, and it also turned out that the derivative (**41**) exhibited a better protective effect in heart damage compared to losartan. It is worth mentioning that the esterification of the losartan primary alcohol group was necessary to achieve the described results because the administration of losartan and dihydrocaffeic acid as not-esterified compounds did not show comparable results [97].

### 6.2. Anti-Inflammatory Activity

The anti-inflammatory properties of dihydrocaffeic acid and its derivatives were confirmed in several studies [98,99,100,101,102]. DHCA together with dihydroferulic and dihydroxyphenylacetic acids were only 3 out of 18 polyphenol compounds that were able to inhibit at least 50% of the prostaglandin E2 formed by colon fibroblast cells (CCD-18) after their stimulation with interleukin-1β (IL-1b) [98]. The same authors acknowledged the anti-inflammatory activity of these three compounds both with in vitro and in vivo methods. When DHCA was given orally to rats with the dextran sodium sulfate-induced colitis, decreased weight loss and water content in faeces were observed in comparison with rats without this phenolic acid. RT-PCR investigations of the expression of several cytokines in rat distal colon mucosa revealed that DHCA reduced the expression levels of IL-1b, IL-8, and TNF-α. Additionally, DHCA diminished DNA damage and malondialdehyde levels [98]. Similarly, dihydrocaffeic and dihydroxyphenylacetic acids, in concentrations of 1 µM, inhibited the secretion of the main pro-inflammatory cytokines, namely TNF-α, IL-1b, and IL-6, in the lipopolysaccharide (LPS)-stimulated peripheral blood mononuclear cells (PBMC) of six volunteers [99]. Oppositely, Sánchez-Medina et al. [100] indicated that DHCA did not exhibit anti-inflammatory activity because it did not affect the production of interleukins IL-6 and IL-8 nor chemokines, namely monocyte chemoattractant protein-1 (MCP-1) and macrophage inflammatory protein-1β (MIP-1β), in TNF-α stimulated human hepatoma HepG2 cells [100].

**Figure 9:**
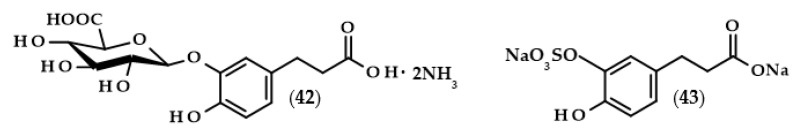
Chemical structures of dihydrocaffeic acid 3-*O*-β-D-glucuronide diammonium salt (**42**) and dihydrocaffeic acid 3-*O*-sulfate disodium salt (**43**).

In recent times, scientists are interested not only in dihydrocaffeic acid but also in its derivatives, especially those formed during its metabolic fate such as glucuronides or sulfates. The reasonability of such a study is understandable, mainly due to the fact that it is necessary to assess which metabolic products actually have antioxidant/anti-inflammatory effects in a living organism. González de Llano et al. [101] compared the effectiveness of mitigating neuroinflammation and oxidative stress using dihydrocaffeic acid and its metabolic derivatives, in the form of dihydrocaffeic acid 3-*O*-β-D-glucuronide diammonium salt (**42**) and dihydrocaffeic acid 3-*O*-sulfate disodium salt (**43**) (Figure 9), as well as two other phenolic acids, i.e., dihydroxyphenylacetic and protocatechuic acids.

The research model constituted human neuroblastoma cells (SH-SY5Y) and murine macrophage cells (RAW 264.7) that were treated with LPS in the case of inducing inflammation, and human cells were also treated with *tert*-butyl hydroperoxide in order to induce oxidative stress. Interestingly, glucuronide derivative (**42**) exhibited the highest cytoprotective effect, significantly attenuated ROS accumulation, and inhibited the production of pro-inflammatory cytokines, confirming its anti-inflammatory and antioxidative properties in the tested cell lines. The parent compound and sulfate derivative (**43**) presented definitely weaker protective effects [101].

The amide derivative of dihydrocaffeic acid and spermine—Kukoamine A (**8**) was also considered to be a substance with antioxidant and anti-inflammatory effects. Despite it being found that it acts as an efficient antioxidant agent in test with DPPH radical and in lipid peroxidation tests with AAPH, as well as exhibiting comparable results with indomethacin in the inhibition of carrageenan-induced rat paw oedema, a high cytotoxic activity was observed when kukoamine A was tested on rat endothelial cells [102].

### 6.3. Cytoprotective Activity

The cytoprotective activities of DHCA and several derivatives have been proven, and special emphasis was held on the mitigation of UV and chemical damage to different cell lines. The term cytoprotection can be understood as the activity of a chemical compound in reducing the cytotoxic and pro-inflammatory effects of selected factors which affect living cells. The effect of the cytoprotective activity of DHCA was studied both in vitro and in vivo. Larrosa et al. [103] evaluated two different concentrations of this phenolic acid (10 and 100 µM) and its mixture with *p*-coumaric acid in a ratio of 1:1 (5 + 5 µM) using human conjunctival cells treated with UVB irradiation (312 nm) of 44 J/m^2^ and in vivo models of rabbit’s cornea and sclera with UVB exposure of 79 J/m^2^. Already, a lower concentration of DHCA and the mixture of two phenolics were able to minimize the destructive effect of UVB irradiation, where the in vitro test revealed that the level of 8-oxo-2′-deoxyguanosine, a marker of oxidative DNA damage, was significantly reduced. When rabbit eyes were treated with DHCA or the mixture of DHCA + *p*-coumaric acid before the UVB application, the levels of pro-inflammatory cytokine (prostaglandin E2), malondialdehyde (a measure of lipid peroxidation), and DNA oxidative damage were diminished. Therefore, the authors indicated the possibility of using these phenolic acids as a topical treatment for UVB damage, due to the satisfying results of the in vivo attempts [103].

Both the positive and negative effects of solar radiation are well known. On the one hand, the synthesis of vitamin D comes to mind, and on the other, excessive radiation can cause inflammation and even cancer. Poquet et al. [104] decided to investigate UVB irradiation and the use of dihydrocaffeic acid act on human keratinocyte HaCaT cells. The application of DHCA before, after, and before and after UV exposure led to a decrease in the concentration of IL-8. Similarly, this phenolic acid reduced IL-6 production, but the results were not dose-dependent. Moreover, in the comparison with the other phenolic acids, it was revealed that DHCA and its unsaturated derivative (caffeic acid) were able to diminish IL-8 levels after UV exposure. In addition to having a catechol ring, compounds with propane/propene tails were significantly more efficient than similar phenolics such as, e.g., ferulic, dihydroferulic, and protocatechuic acids [104].

**Figure 10:**
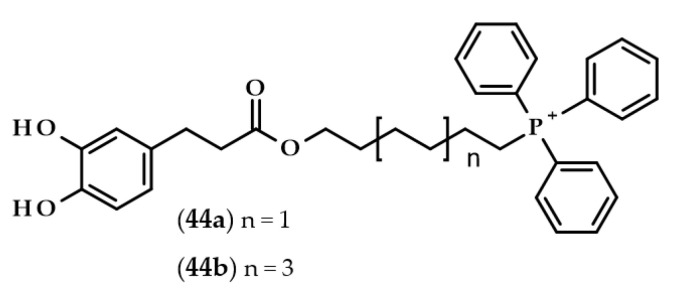
Chemical structures of mitochondriotropic antioxidants: (6-(3-(3,4-dihydroxyphenyl)propanamide)hexyl)triphenylphosphonium methanesulfonate (**44a**) and (10-(3-(3,4-dihydroxyphenyl)propanamide)decyl)triphenylphosphonium methanesulfonate (**44b**).

Completely different derivatives were developed and tested by Benfeito et al. [105]. Dozens of synthesized compounds consisted of a carboxamide with a phenolic ring, 6- or 10-carbon chain alkyl linker, and triphenylphosphonium moiety, which is a well-known mitochondrial targeting vector. Some of the compounds had dihydrocaffeoyl moiety, namely, (6-(3-(3,4-dihydroxyphenyl)propanamide)hexyl)triphenylphosphonium methanesulfonate (**44a**) and (10-(3-(3,4-dihydroxyphenyl)propanamide)decyl)triphenylphosphonium methanesulfonate (**44b**) (Figure 10).

All of the compounds exhibited antioxidant activities. However, compound **44b** exerted cytotoxic properties at the concentration of 50 μM on SH-SY5Y cells and at an even lower concentration, i.e., 10 μM, when evaluated with HepG2 cells. Interestingly, compound **44a**, which was the compound with the shorter linker (6 carbons instead of 10), did not show any cytotoxicity at all. Furthermore, compound **44a** was also one of the several compounds that were able to cross an in vitro blood–brain barrier model, as well as protecting SH-SY5Y cells from oxidative damage [105].

The protective properties against toxic compounds were also evaluated not only for dihydrocaffeic acid but also for tungtungmadic acid (3-caffeoyl-4-dihydrocaffeoyl quinic acid, (**3**) [21,22,23,106]. In the case of DHCA, both compounds with urolithin B exhibited a potent increase in the survival of the human neuroblastoma SK-N-MC clonal cell line with oxidative stress induced by 2,3-dimethoxy-1,4-naphtoquinone [106]. The latter, tungtungmadic acid, diminished the generation of ROS in tert-butyl hydroperoxide-induced Hepa1c1c7 liver cells, inhibited the activation of caspase-3 (a significant protein of the execution phase of cell apoptosis), and up-regulated heme oxygenase-1 expression, an enzyme with potent antioxidant and anti-inflammatory activities [21]. This compound, isolated from *S. herbacea*, exhibited a high antioxidant activity and inhibited lipid peroxidation and chemical-induced DNA strand breaks [23]. The authors also confirmed that the administration of compound **3** to mice reduced inflammation and acute carbon tetrachloride-induced hepatic fibrosis [22].

Several papers have also evaluated the opposite activity of these compounds, i.e., cytotoxic activity. One of the examples mentioned before, namely kukoamine A, exerted high cytotoxic activity with IC_50_ of 3.5 µM on rat endothelial cells [102]. Lee et al. [107] have shown that phenolic compounds, especially those containing a catechol ring, can possess both antioxidant and pro-oxidant properties. Cytotoxic activities of DHCA on HCT 116, INT 407, and IEC-6 cells were observed in concentrations ranging from 232 to 312 µM. According to the authors’ findings, the main factor that is responsible for the cytotoxic effects of DHCA is the generation of ROS [107].

### 6.4. Anticancer Activity

**Figure 11:**
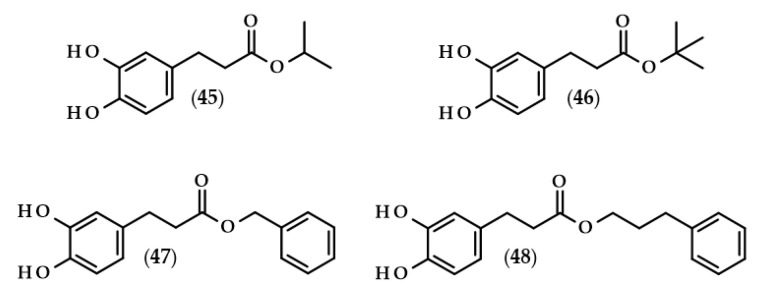
Dihydrocaffeic acid derivatives evaluated as anticancer agents. Chemical structures of: isopropyl dihydrocaffeate (**45**), *tert*-butyl dihydrocaffeate (**46**), benzyl dihydrocaffeate (**47**), 3-phenylpropyl dihydrocaffeate (**48**).

With reference to the last paragraph of the previous subsection, the anti-cancer effect is really nothing more than a cytotoxic effect on cells, and, more specifically, on cancer cells. It is extremely important to properly screen chemical compounds in the search for new potential anticancer drugs so that they show a specific target and do not have a toxic effect on non-cancer cells. Several papers have regarded the anticancer activity of DHCA and its esters (Figure 11) on different cell lines [108,109,110,111,112]. Summarized data of IC_50_ values, that is the concentration that decreases cell viability by 50%, are presented in Table 3

Probably the first such extensive studies on the assessment of the toxicity of 10 esters of caffeic and dihydrocaffeic acids on two cell lines (mouse leukemia cell line L1210 and breast cancer cell line MCF-7) along with the extended elaboration of quantitative structure-activity relationships (QSAR) models were carried out by Etzenhouser et al. [108]. The authors synthesized both linear alcohol esters of DHCA: methyl (**2**), ethyl (**17**), butyl (**19**), hexyl (**20**), and octyl (**21**) esters; branched alcohol derivatives: isopropyl (**45**) and *tert*-butyl (**46**) esters; and aromatic alcohol dihydrocaffeates: benzyl (**47**), phenethyl (**25**), and 3-phenylpropyl (**48**) conjugates. The chemical structure of the tested esters played a pivotal role in exerting anticancer activities. For both cancer lines, cytotoxic activities were observed, but MCF-7 cells were more resistant. The IC_50_ values ranged from 0.009 to 0.024 mM in the case of L1210 cells, and from 0.050 to 0.132 mM for MCF-7 cells (Table 3). It has been shown that octyl dihydrocaffeate (**21**) was the compound which exhibited the highest cytotoxic activities on both cell lines. Comparable anticancer activity was also observed for hexyl dihydrocaffeate (**20**) and *tert*-butyl ester (**46**) when they were tested on L1210 and MCF-7 cell lines, respectively. The authors proved also that the majority of corresponding caffeic acid esters exhibited significantly higher anticancer activities, but only when they were investigated with the L1210 cells [108].

Gomes et al. [109] conducted a comparative study of selected phenolic acids in terms of their antiproliferative and cytotoxic properties. Among the tested compounds, both di- and trihydroxylated phenolics were evaluated, and four cell lines were applied, i.e., L-132 (human lung epithelial cell line), HeLa (human cervix carcinoma cells), MDA-MB-231 (mammary gland adenocarcinoma cells), and MOLT-3 (lymphoblastic leukemia cells). Regarding dihydrocaffeic acid, it was found that this compound exhibited antiproliferative properties, mainly on HeLa cells, and acted moderately as an anticancer drug on the tested cells, and the cell viabilities after 72 h of incubation with DHCA in the concentration of 100 µM were at least 70%. The authors showed that phenolics with three hydroxyl groups in the aromatic ring were more potent anticancer drugs, and when DHCA was compared with its unsaturated derivative, it was proven that the double bond increased the cell viability and thus caffeic acid exhibited lower antiproliferative and anticancer properties than dihydrocaffeic acid [109].

The anticancer activity of dihydrocaffeic acid was also assessed by Kudugunti et al. [110] and Vázquez et al. [111]. The IC_50_ value of 2.2 mM after 48 h was indicated with the human melanoma cell line (SK-MEL-24) [110]. Interestingly, the effect of the action of DHCA within the U-937 leukemic cells was dependent on the concentration of the phenolic acid, and the authors observed that the concentration of 0.212 mM induced a 50% cell proliferation, and that the concentration that decreased cell viability by 50% was higher than 2 mM [111].

A totally different application of dihydrocaffeic acid in anticancer therapy was discovered by Zhao et al. [112]. Right now, the possible anticancer effect of this acid is not crucial, but rather the presence of a catechol ring. In this paper, DHCA-hyaluronic acid conjugates served as linkers for gold nanoparticles (AuNCs). Furthermore, such scaffolds were also enriched in Raman reporter (2-naphthalenethiol) and a pheophytin derivate—pheophorbide-a. Subsequently, modified nanoparticles were applied in synchronous cancer detection and photodynamic therapy. Nearly complete cellular uptake in HeLa cells and high phototoxicity even at low concentrations of pheophorbide-a were observed for the obtained AuNC conjugates. The presence of the conjugates in cancer cells was proven by means of Raman spectroscopy, TEM and confocal microscopy, and flow cytometry. The laser exposure time and the ratio of DHCA to hyaluronic acid, and thus the number of attached nanoparticles, played an essential role in the decreasing of the HeLa cells’ viability, where after 10 min of phototherapy, over 80% of the cells died [112]. The conducted research was inspired by natural phenomena, and more specifically the adhesion phenomenon which occurs in mussels. The adhesive proteins secreted by muscles have amino acids with catechol rings that affect the strength of adhesion. The subject of the use of dihydrocaffeic acid in the design of adhesive gels with, e.g., chitosan and their biomedical and drug delivery applications, are described in Section 6.6. Hydrogels, Resins, and Polymers Preparation for Biomedical Application.

### 6.5. Enzyme Inhibitory Activity

In search of a niche for the acid and its derivatives, researchers also assessed their usefulness in inhibiting the activity of selected enzymes. Enzyme inhibitors are often used in medicine; therefore, many substances of natural origin are tested in this respect. An example of such substances may be inhibitors of interleukin-2 inducible T-cell kinase, i.e., tyrosine kinases mainly expressed in CD4^+^ T-cells, which are used in the treatment of different inflammatory disorders. This was also the case for the metabolites isolated from the flowers of the Australian rainforest tree (*P. murrayi*). Dihydrocaffeic acid, as one of the obtained compounds, exhibited a low IC_50_ value of 418 µM; its analogue with one hydroxyl group exerted at least six-fold higher activity as the inhibitor of tyrosine kinase (64 µM), and the highest activity was observed for a mixture of isomers of 4-hydroxyphenylpropionic and quinic acid derivatives (58 µM) [9].

Thirty-three carboxylic acid derivatives, both linear and branched, and cyclic and with the aromatic ring bearing various substituents, were compared as potential histone deacetylase inhibitors. The inhibition of the deacetylation of histones may lead to a lag in the growth and differentiation, and even to apoptosis; therefore, such inhibitors are sought after. DHCA caused partial inhibition of the evaluated enzyme and 86% of the remaining activity of histone deacetylase was still observed. According to the authors’ recommendation, if the activity was above 85%, the tested compound was less active than sodium butyrate, a reference inhibitor. The authors, amongst all 33 chemicals, claimed that caffeic and ferulic acids, as well as their derivatives, i.e., chlorogenic acid and curcumin, were the most active compounds, with significantly higher inhibitory properties towards deacetylase than sodium butyrate [113].

Chen et al. [114] used a molecular dynamics simulation approach to validate whether several compounds of natural origin may act as inhibitors of insulin-degrading enzyme. DHCA had the highest docking score among all of the evaluated compounds. The compounds affected the insulin-degrading enzyme, probably through competitive binding and steric hindrance. Thanks to the virtual screening of the compounds, the authors showed that DHCA formed an H-bond interaction with the key amino acid residue Asn139, and through the zinc ion (an ion necessary for the functioning of this enzyme), was connected to the active pocket. Therefore, such interactions with these compounds may limit insulin degradation [114].

**Figure 12:**
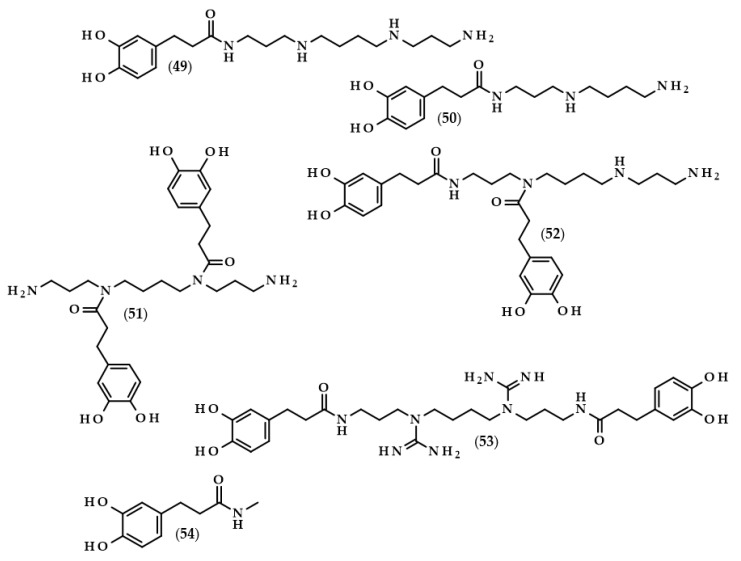
Dihydrocaffeic acid derivatives evaluated as enzyme inhibitors: N^1^-(dihydrocaffeoyl)spermine (**49**), N^1^-(dihydrocaffeoyl)spermidine (**50**), Kukoamine C (N^4^,N^8^-*bis*(dihydrocaffeoyl)spermine) (**51**), Kukoamine D (N^1^,N^4^-*bis*(dihydrocaffeoyl)spermine) (**52**), N^1^,N^12^-*bis*(dihydrocaffeoyl)-N^4^,N^8^-*bis*(guanidyl)spermine (**53**), and 3-(3,4-dihydroxyphenyl)-*N*-methylpropanamide (**54**).

Kukoamine A (**8**) and other acylpolyamines, both of natural origin, and those obtained synthetically, were also evaluated as enzyme inhibitors. One of the targets of these potential drugs has become trypanothione reductase (TR) from *Crithidia fasciculate* parasites. TR, similar to glutathione reductase, is involved in protecting against oxidative damage. Ponasik et al. [115] have reported the synthesis of several acylpolyamines, as well as those that had dihydrocaffeyol moieties. Kinetic studies with TR in the presence of the synthesized compounds were then performed. It was revealed that kukoamine A (N^1^,N^12^-*bis*(dihydrocaffeoyl)spermine) (**8**) and N^1^,N^8^-*bis*(dihydrocaffeoyl)spermidine (**10**), as well as their analogues without one dihydrocaffeoyl moiety, namely N^1^-(dihydrocaffeoyl)spermine (**49**) and N^1^-(dihydrocaffeoyl)spermidine (**50**) (Figure 12), were competitive inhibitors of TR. Kukoamine A (**8**) was found to be the most active compound, with a kinetic constant of K_I_ = 1.8 µM and K_II_ = 13 µM. Spermidine derivative of dihydrocaffeic acid (**10**) was a slightly less potent inhibitor (K_I_ = 7.5 µM). Comparing polyamines by the number of dihydrocaffeyol moieties, those with only one catecholic ring had inhibitory properties which were roughly 50- and 15-fold lower than their analogues, and the K_I_ for compound **49** was 85 µM, and for compound **50**, K_I_ was 108 µM. The authors proved that two catecholic rings were crucial for high activities, and the presence of an unsaturated bond in the carbon chain of phenolic acid reduced the activity [115].

Lipoxygenases were another target for kukoamines, which are involved in the biosynthesis of leukotrienes from arachidonic acid and in the production of hydroperoxides in the lipid bilayer. Kukoamine A (**8**) exhibited high inhibitory activity towards soybean lipoxygenease and the IC_50_ value was 9.5 µM. The authors also assessed DHCA and its mixtures in the ratio of 2:1 (phenolic acid:polyamine), that is, DHCA:spermine and DHCA:spermidine, as well as kukoamine B (**9**), kukoamine C (**51**), kukoamine D (**52**), and one synthetic derivative—N^1^,N^12^-*bis*(dihydrocaffeoyl)-N^4^,N^8^-*bis*(guanidyl)spermine (**53**). All of the mentioned compounds or their mixtures did not exhibit lipoxygenase inhibition as high as that of kukoamine A, and their percentage inhibition ranged from 5.4% to 32% [116].

The angiotensin-converting enzyme (ACE) inhibitory activity was confirmed for compound **10**, with an IC_50_ value of 9.55 ppm. This value was almost 75 times lower than the inhibitory activity of lisinopril (709.36 ppm), a medication and a well-known ACE inhibitor. During in vitro tests, the ACE inhibition was also observed for fresh fruit pulp of lulo (*S. quitoense*, 1.08 ppm), as well as for freeze-dried (83.49 ppm) and spray-dried fruits (43.14 ppm), where the authors indicated that, probably, it was not only compound **10** that had inhibitory activity [31].

Among other dihydrocaffeic acid derivatives, (3α,21β)-Lycophlegmariol A (**13**) isolated from *H. phlegmaria*, along with other metabolites of this fern, were evaluated as acetylcholinesterase (AChE) inhibitors. Only two out of the six tested compounds exerted AChE inhibition, and (3α,21β)-Lycophlegmariol A (**13**) had no activity towards this enzyme [33].

Enzyme inhibitors may also find use in the treatment of bacterial infections. In the research of Allegretta et al. [117], new inhibitors were sought for the PqsD enzyme from *P. aeruginosa*, which is a key protein in the quorum sensing (QS) of this bacterium. Of the 25 compounds which were evaluated, 6 of them were derivatives of dihydrocaffeic acid, and these were: DHCA (**1**), methyl dihydrocaffeate (**2**), ethyl dihydrocaffeate (**17**), isopropyl dihydrocaffeate (**45**), benzyl dihydrocaffeate (**47**), and 3-(3,4-dihydroxyphenyl)-*N*-methylpropanamide (**54**). The findings of the authors showed that compounds bearing catechol rings and with at least two-carbon saturated linkers exhibited higher inhibition activities compared with the other chemicals. It has also been indicated that the ester moiety was also an important feature of the tested compounds, and the IC_50_ values ranged from 5.9 to 27 µM and decreased in the following order: DHCA (**1**) > methyl ester (**2**) > ethyl ester (**17**) > isopropyl ester (**45**) > benzyl dihydrocaffeate (**47**). Regarding the amide derivative of DHCA, compound **54** exerted a similar activity (IC_50_ = 20 µM) to DHCA and its methyl ester [117].

### 6.6. Hydrogels, Resins and Polymers Preparation for Biomedical Application

This subsection does not fully address the specific properties of dihydrocaffeic acid, but rather discusses the use of this substance to obtain hydrogels, such as those previously mentioned with hyaluronic acid [112] or chitosan [118,119,120,121,122,123], as well as resins and polymers that can be used for biomedical application or construction engineering [124,125,126,127,128].

Researchers, inspired by mussels, use DHCA or other similar compounds and a biopolymer—chitosan for the production of adhesive hydrogels. Xu et al. [118] prepared such chitosan hydrogels with the use of DHCA, L-dopa, or dopamine. The obtained hydrogels were compared by means of swelling, mucoadhesive to rabbit small intestine, and the release of compounds with the catechol ring. In the presence of dihydrocaffeic acid, swelling properties were lower compared to the rest of the compounds, and the slowest catechol release was observed. Furthermore, these features increased mucoadhesive properties in the rabbit intestine, which was about two-fold better in comparison to pristine chitosan gel [118].

Possibilities for the adsorption of dyes on the chitosan surface have been improved by using the tyrosinase-mediated grafting of phenolic acids to chitosan. This polysaccharide is known for its ability to adsorb dyes, although not those of cationic character. Chao et al. [120] showed that 4-hydroxybenzoic, 3,4-dihydroxybenzoic, 3,4-dihydroxyphenylacetic, and dihydrocaffeic acids were conjugated individually with the chitosan and exhibited adsorption of two cationic dyes in batch experiments, namely crystal violet and bismarck brown Y dyes. Among the used phenolic acids, DHCA was found to be the most efficient in the adsorption processes, mainly due to belonging to the most flexible carboxyl group, that is, having the longest carbon side chain in comparison with the rest of the phenolics. What is also important, the modified chitosans were still able to adsorb anionic dyes, such as amaranth, which meant that protonated amine groups were still active [120].

The purified laccase from *C. unicolor* was used for the functionalization of chitosan, and C-N bond formation was observed between dihydrocaffeic or protocatechuic acids and the amine group of glucosamine. Prolonged incubation of the reaction mixture with the laccase enzyme resulted in a significant weakness of the intended activity, due to the quinone formation from the oxidizing of the catechol ring. The obtained DHCA-chitosan hydrogels were highly crosslinked and exhibited moderate adhesion activity. In the case of the second phenolic acid, protocatechuic acid-chitosan agglomerates were formed, and their adhesion strength was at least two-fold higher than that obtained for gum arabic [121].

In the study conducted by Kim et al. [122], it was shown that chitosan was modified with 1-ethyl-3-(3-dimethylaminopropyl)-carbodiimide hydrochloride and DHCA molecules were joined to amine residues. Such an approach allowed for obtaining highly water-soluble modified chitosan with a solubility of 60 g/L at pH = 7. In addition, strong adhesive properties have been demonstrated, which can translate into the use of such hydrogels in biomedical applications [122].

A certain modification regarding the preparation of hydrogels was introduced by Liang et al. [123]. Besides the formation of DHCA-chitosan hydrogel, the authors also prepared pullulan oxidized by NaIO_4_ and, finally, combined both modified polysaccharides (CS-DA/OP) via a Schiff base reaction; thus forming -C=N- bonds. The resulting CS-DA/OP hydrogels were thoroughly characterized by means of their morphology, rheological properties, and anticancer and antibacterial activities after the encapsulation of doxorubicin or amoxicillin, respectively. This study indicated that the obtained hydrogels had satisfactory mucosal adhesive properties, the release of doxorubicin was pH-dependent, and that they were successful in limiting the viability of the HCT116 cancer cells. Moreover, acceptable antibacterial activities towards *E. coli* and *S. aureus* were observed after the encapsulation of amoxicillin [123].

Chitosan was not the one and only polymer that was used for the preparation of adhesive hydrogels. Brubaker and Messersmith’s approach [124] was based on the Ala-Ala dipeptide, a branched poly(ethylene glycol) and dihydrocaffeic acid. The presence of dipeptides in the structure of the hydrogel increased its enzymatic biodegradability because Ala-Ala is a substrate for neutrophil elastase. Studies performed in vivo, resulting from subcutaneous implantation of the obtained hydrogel in mice, demonstrated a slight inflammatory reaction of the tissue and slow degradation of the preparation was confirmed [124].

Moving on to the field of materials and engineering science, compounds bearing catechol moieties found use in the modification of perovskite solar cells, which are considered to be a major breakthrough technology, and intensive development of research on their use for electricity production is underway. Catechols, such as 3,4-dihydroxyphenylalanine, 3,4-dihydroxyphenethylamine, and DHCA, can serve as linkers in the buried interface between perovskite via amino and/or carboxyl group and tin(IV) oxide via hydroxyl groups from the catechol ring. The authors indicated enhanced electrical properties of the modified solar cells. Moreover, the power conversion efficiencies of all of the obtained cells were higher than the control and were increasing in the following order: DHCA < 3,4-dihydroxyphenethylamine < 3,4-dihydroxyphenylalanine [125].

Remaining in the engineering sciences, Sun et al. [126] designed and synthesized DHCA-modified epoxy resins with potential applications in construction engineering for covering and protecting the concrete substrate. The adhesive strength increased with increasing of the dihydrocaffeic acid content in modified epoxy resins. The main factor that influenced the adhesive properties was the formation of hydrogen bonds and electrostatic interactions between hydroxyl groups from the aromatic ring and saturated concrete substrate. Interestingly, 120 days of immersion of the epoxy resins with 20% of DHCA in water caused only a 22% decrease in the strength of adhesion [126].

Crosslinked polyethylenimine-DHCA nanogels with an average diameter of 111 nm were obtained and adopted as small interfering RNA (siRNA) carriers by Kim et al. [127]. The combination of the primary amino groups with the hydroxyl group of DHCA resulted in the formation of stable complexes with siRNA, in which cellular uptake in MDA-MB-435 cells was boosted compared to the control gels without this phenolic acid. Moreover, complexes of polyethylenimine-DHCA-siRNA also exhibited lower cytotoxicity towards the evaluated cells than other tested preparations, which indicates their possible use as siRNA carriers [127].

The last but not least possible application of DHCA was described by Jiang et al. [128]. The authors presented the preparation of carbon dots, which were designed from gadolinium chloride, DHCA, and 2,2′-(ethylenedioxy)*bis*(ethylamine), and exhibited good biocompatibility in cell cultures and in mice. The nanostructure, designed in this way, has been enriched with doxorubicin hydrochloride and IR825 (a near-infrared dye) and used in the combined photothermal chemotherapy for triple-negative breast cancer. A high chemotherapy efficiency was noticed as well as the confirmation of magnetic resonance imaging ability. Through the application of the fabricated nanostructure, the near-infrared light was converted to heat, resulting in the inhibition of tumor growth [128].

### 6.7. Antimicrobial Activity

Studies of the antimicrobial activity of phenolic compounds, apart from their antioxidant activity, are the most frequently undertaken research topics in the search for the biological activity of single compounds, extracts of natural origin, or (chemo)enzymatically obtained derivatives. One report on the inhibition of microbial growth by phenolic compounds specifically concerned the disintegration and destabilization of the outer membrane of three strains of *Salmonella enterica*. Several mono- and di-hydroxy phenolic acids were evaluated and, among them, DHCA affected the destabilization of the outer membranes of *S. enterica* subsp. *enterica* serovar Typhimurium and *S. enterica* subsp. *enterica* serovar infantis, which was observed through increased uptake of the fluorescent probe. The authors revealed that these compounds interacted with divalent cations found in the membrane and thus decreased its stabilization. Furthermore, the application of dihydrocaffeic acid increased the susceptibility of *S.* Typhimurium strains to novobiocin and, in the case of *S.* Infantis, about an 11% LPS release was observed when 2.5 mM of DHCA was added to the bacterial cultures [129].

The antimicrobial activities of 13 phenolic acids and 2 flavan-3-ols towards commensal, probiotic, or pathogenic bacteria and yeast were reported by Cueva et al. [130]. Non-substituted compounds such as benzoic acid, as well as *meta*- and *para*-hydroxylated derivatives, i.e., 3-hydroxybenzoic, 4-hydroxybenzoic, and 4-hydroxyphenylpropionic acids, were the most potent phenolic acids, with the highest inhibitory activities against the tested microorganisms. Depending on the evaluated strains, the compounds with a catechol ring exhibited moderate or low antimicrobial properties. In the case of DHCA, a concentration of 1 mg/mL allowed it to inhibit 33% of *L. paraplantarum* LCH7, and roughly 41% of *S. aureus* EP167. Approximately 35 and 50% of inhibition growth was observed towards *E.coli* strains [130].

It has also been proven that dihydrocaffeic acid reduced biofilm formation in *P. aeruginosa* PAO1. A biofilm inhibition rate of 19.82% was noted for DHCA, while caffeic acid and azithromycin (positive control) were observed to have rates of 22.67 and 35.31%, respectively [131]. The research on dihydrocaffeic acid was part of a larger study on the effects of natural substances on *P. aeruginosa* and other bacteria’s growth and biofilm formation [132]. It should be noted that DHCA, along with quinic acid, were able to limit biofilm formation by *P. aeruginosa* PA1803 and, moreover, DHCA and gallic acid affected the integrity of the outer membranes of *P. aeruginosa* and *S. aureus* strains. The antibacterial activity of dihydrocaffeic acid was found to be 33 times more potent than that of chlorogenic acid against *S. aureus*, and in the case of *P. aeruginosa*, neither DHCA nor other tested compounds affected its growth [132].

DHCA and butyl dihydrocaffeate (**19**) were examined in terms of antimicrobial activities towards three Gram-positive bacteria, three Gram-negative bacteria, and one fungal strain. The minimum inhibitory concentrations for both compounds ranged from 2 to 16 mM, except for one species of fungi. DHCA did not inhibit the growth of *Rhizopus oryzae* DSM 2199, while significantly greater antifungal activity was observed when its butyl ester was applied. The minimum inhibitory concentration (MIC) and minimum microbicidal concentration (MMC) were 1 and 2 mM, respectively, which confirms the validity of the lipophilization of phenolic compounds and the boosting of their biological activities [1].

**Figure 13:**
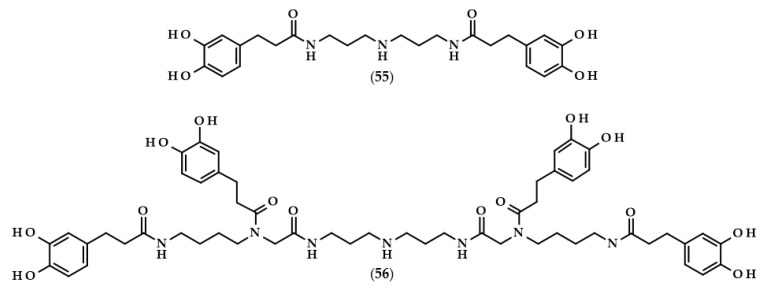
Structures of dihydrocaffeoyl–polyamines evaluated as antibacterial compounds: N^1^,N^8^-*bis*(dihydrocaffeoyl)norspermidine (**55**) and *tetra*(dihydrocaffeoyl)polyamine conjugate (**56**).

Yingyongnarongkul et al. [133] undertook the solid-phase synthesis of (dihydrocaffeoyl)polyamine conjugates. The six obtained compounds (Figure 13), i.e., N^1^,N^8^-*bis*(dihydrocaffeoyl)norspermidine (**55**), Kukoamine A (N^1^,N^12^-*bis*(dihydrocaffeoyl)spermine) (**8**), N^1^,N^8^-*bis*(dihydrocaffeoyl)spermidine (**10**), N^1^,N^4^,N^12^-*tris*(dihydrocaffeoyl)spermine (**11**), and *tetra*(dihydrocaffeoyl)polyamine conjugate (**56**) were compared in regards to their antibacterial activity with the use of 11 strains of MRSA (methicillin-resistant *S. aureus*) and 4 VRSA (vancomycin-resistant *S. aureus*). The anti-*staphylococcal* activity of the acylated polyamines was independent of the resistance mechanism detected in the tested strains. All compounds which were obtained, except for compound **56,** gave average MIC values of 25–50 μg/mL, but they were better than those for a reference drug—vancomycin in VRSA strains. *Tetra*(dihydrocaffeoyl)polyamine conjugate (**56**) exerted the highest antibacterial activities, which showed that the higher the number of dihydrocaffeoyl moieties, the higher the antimicrobial activity. It is also worth noting that the synthesized compounds did not affect the growth of the Vero cells with IC_50_ values above 50 μg/μL [133].

### 6.8. Antiviral Activity

In the scientific literature, there are also reports on the antiviral activity of dihydrocaffeic acid and its derivatives. The first one was published in 1991 and describes the activity of humic acids and humic-acid-like polymers, synthesized from various phenolic acids, against *herpes simplex virus* type 1 (HSV-1) [134]. The authors claimed that polymers of the following phenolics: gentisic, caffeic, dihydrocaffeic, and 3,4-dihydroxyphenylacetic acids inhibited virus multiplication with a minimum effective concentration (MEC) in the range from 2 to 10 µg/mL and maximum tolerated concentration (MTC), referring to host cells, from 40 to 160 µg/mL. In the case of dihydrocaffeic polymer, the specific values of MEC and MTC were 10 and 160 µg/mL, respectively, which means that it can be a potent HSV-1 inhibitor and, among the described compounds, host cells tolerated its highest concentration [134].

The research on the antiviral activity of lipophilic catechols was also conducted by Italian teams, which evaluated enzymatically obtained compounds against influenza A virus [70], as well as other RNA viruses, such as Poliovirus type 1, Echovirus type 9, Coxsackievirus type B3 (Cox B3), and the following DNA viruses: HSV-1, HSV-2, Adenovirus type 2 and type 5, and Cytomegalovirus (CMV) [71]. Methyl (**2**), ethyl (**17**), propyl (**18**), and butyl (**19**) esters of dihydrocaffeic acid did not affect the replication of the influenza A virus, but interestingly, their analogues—esters of short carboxylic acids and hydroxytyrosol as a phenolic part, namely hydroxytyrosyl butyrate and hydroxytyrosyl pentanoate, exhibited high activity [70]. In the case of other viruses, butyl dihydrocaffeate (**19**) was active against Cox B3 and CMV with IC_50_ values of 100 and 25 µg/mL, respectively, and, in turn, ethyl ester (**17**) also inhibited the replication of HSV-1 and HSV-2 with 15 and 60 µg/mL for 50% inhibition of virus plaque formation [71].

### 6.9. Repellent Activity, Avoidance Response in Caenorhabditis elegans and Insecticidal Activity

Dihydrocaffeic acid is considered to be a water-soluble repellent. DHCA was used in research on avoidance response in *Caenorhabditis elegans*, the model organism and an extensively studied nematode. Aoki et al. [135] identified a seven-transmembrane receptor, DCAR-1 (dihydrocaffeic acid receptor), which was defined as a receptor required for avoidance of the aforementioned repellent. For the survival of not only nematodes but also other animals, a rapid detection of harmful chemicals is very important. In the case of *C. elegans*, the response to various repellents is associated with their moving away from chemicals. The authors evidenced that *dcar-1* mutant nematodes, hence those without the described receptor, were defective in the avoidance response. The research was continued by Zugasti et al. [136], and the authors suggested that DCAR-1 was required for the response to fungal infection of the endoparasitic fungus *Drechmeria coniospora* and wounding. Furthermore, dihydrocaffeic acid acted as an exogenous ligand in the activation of the innate immune response through the DCAR-1 receptor. The findings of the above-cited authors allow for a better understanding of the relevance and chemosensory signal transduction in the avoidance response to DHCA and other phenolic repellents in *C. elegans*.

In the research of Fraga et al. [6] the insecticidal activity of DHCA was demonstrated. The authors isolated 35 different compounds from the methanolic extract of *N. teydea* roots and 16 chemicals or their mixtures were compared in antifeedant and oral toxicity tests to *Spodoptera littoralis* and cytotoxic activities towards pupal ovarian tissue cells of *Spodoptera frugiperda* (Sf9 cells). Dihydrocaffeic acid was proven to be a strong toxicant that affected larval weight and consumption. As well, strong cytotoxic properties were observed against Sf9 cells, where the ED_50_, thus the concentration required for 50% cell viability, was 14.4 µg/µL [6]. 

### 6.10. The Complexation of Metals by Dihydrocaffeic Acid

Fulvic and humic acids, as well as humin, are mixtures of macromolecular organic compounds included in soil humus. The vast majority of chemical structures that can be distinguished in the humic substances are phenolics. Compounds bearing both catecholic and carboxylic moieties exhibit complex properties and, thus, can increase the bioavailability of different metals for plants [137]. The complexes of copper(II), nickel(II), cobalt(II), iron(III), vanadium(V) and vanadium(IV, V), aluminium(III), chromium(III), and scandium(III) with dihydrocaffeic acid have been documented [138,139,140,141,142,143,144].

The metal-DHCA complexes prepared by Petrou and Koromantzou [138] were dark green (with Co(II) and Ni(II)), dark brown (Cu(II)), or black (Fe(III)). Interestingly, three of them, namely Cu(II), Ni(II), and Fe(III) complexes, were obtained in a 1:1 ratio with the ligand, where the oxygens from the phenolic groups, as well as the chlorine atom, took part in the bonding of the central atom. The complex of Co(II)-DHCA was formed in a 1:2 ratio and only oxygen atoms participated in the complex formation [138].

The research on metal-DHCA complexes was continued by Petrou [139], and binuclear complexes of dihydrocaffeic acid with vanadium(V) and vanadium(IV, V) were studied. Due to the fact that vanadium is an essential element for the human being and may be involved in the inhibition of the sodium-potassium ATPase, while catecholamines, such as norepinephrine, can activate this enzyme, the author decided to apply different spectroscopic, magnetic, and thermogravimetric methods in order to investigate the possibility of forming these complexes. According to the author, the anions of dihydrocaffeic acid, as well as caffeic and ferulic acids, were the ligands in the complexation of vanadium with different oxidation states. Likewise, in the previous work, the catechol ring was crucial for the formation of complexes, in other words, DHCA acted as a bidentate ligand [139].

Another equally important way of using dihydrocaffeic acid is the complexation of toxic metals. Such an element can be aluminum, which is neurotoxic and associated with dementia and Alzheimer’s disease [140,142]. In plants, chromium, in a higher concentration, may also be toxic. The complexation of chromium(III) by DHCA was studied by Petrou et al. [141]. The authors pointed out that the reaction between the metal and the ligand involved a three-step mechanism. Firstly, Cr(III) is bonded by the carboxylic group, then isomerisation is observed, and chelation, as the last step, takes place with the catechol-type of coordination [141]. Last but not least is the element copper, which also exhibits plant toxicity. As was stated above, humic substances are responsible for increasing the bioavailability of some elements, but also for the detoxification processes of other ones. Ligand formation between Cu(II) and dihydrocaffeic acid was investigated by Borges et al. [144], and it was acknowledged again that the presence of a catechol ring was decisive for the preparation of chelates.

### 6.11. Other Biological Properties of Dihydrocaffeic Acid and Its Derivatives

The last subsection of this paper is devoted to the other not-before mentioned biological properties of DHCA and its derivatives, such as their lipid-lowering [145], anti-diabetic properties [146] and inhibitory effect on amyloid aggregation [147,148,149]. Dihydrocaffeic acid, alongside two other hesperetin metabolites, i.e., *m*-hydroxycinnamic and ferulic acids, was shown to be an efficient lipid-lowering compound in rats. Hypolipidemic properties for all of the tested compounds were found in male rats on a high-cholesterol diet (1% *w*/*w*), which was determined based on the significantly lowered total cholesterol and total triglyceride concentrations in plasma compared to the control group. Moreover, the activities of two hepatic enzymes: 3-hydroxy-3-methyl-glutaryl-coenzyme A reductase (HMG-CoA reductase) and acyl-CoA:cholesterol acyltransferase, i.e., key enzymes in the biosynthesis and metabolism of cholesterol, were also remarkably lower than the activities of the enzymes of the rat control group [145].

**Figure 14:**
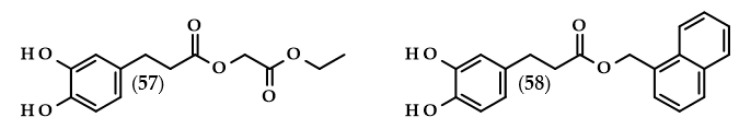
Structures of 2-ethoxy-2-oxoethyl dihydrocaffeate (**57**) and naphthalen-1-ylmethyl dihydrocaffeate (**58**).

A dozen dihydrocaffeic acid derivatives were synthesized by Kim et al. [146]. These compounds were compared for glucose transport activity in the isolated rat epididymal adipocytes that were treated with the obtained compounds for 4 h. The authors observed the highest activities for DHCA and two derivatives: 2-ethoxy-2-oxoethyl dihydrocaffeate (**57**) and naphthalen-1-ylmethyl dihydrocaffeate (**58**) (Figure 14). Among the three most effective compounds, only compound **58** notably decreased the blood glucose levels in streptozotocin-induced diabetic C57BL/6 mice. Additionally, the anti-diabetic activity of naphthalen-1-ylmethyl dihydrocaffeate (**58**) was revealed via the translocation of GLUT4 (glucose transporter 4) in adipocytes, resulting in an increased glucose uptake, and this ester took part in the enhanced clearance of peripheral glucose, as well as acting as a glucose-lowering drug without the use of insulin [146].

Amyloids are protein aggregates which are characterized by a fibrillar morphology, and in the human body are associated with the development of various diseases, such as Alzheimer’s disease and type 2 diabetes. Cheng et al. [147] evaluated whether major coffee components (chlorogenic and caffeic acids or caffeine), as well as the major metabolite—dihydrocaffeic acid, were able to inhibit the amyloidogenicity of human islet amyloid polypeptide (hIAPP). The authors indicated that the polyphenolic structure was necessary to observe the amyloid inhibitory effects; therefore, DHCA showed high activity and inhibited the oligomerization of hIAPP [147]. Compounds isolated from the dried root bark of *L. chinense* kukoamines A (**8**) and B (**9**), and their parent compound DHCA, were examined for their inhibitory effect on the aggregation of amyloid β (Aβ) and hIAPP; thus, meeting therapeutic targets in Alzheimer’s disease and type 2 diabetes [148]. Once again, it was confirmed that the presence of a catechol ring prevented the aggregation of an amyloid, whereas spermidine, as an example of a polyamine, did not exhibit inhibitory effects. The amyloid aggregation was evaluated by thioflavin-T fluorescence assay (Th-T), and it was found that the following order allowed for obtaining the most active compounds: kukoamine B (**9**) > kukoamine A (**8**) > DHCA. In the case of Aβ, the IC_50_ values ranged from 9.5 to 17.2 μM and those for hIAPP were 3.3–9.3 μM. Interestingly, comparing the DHCA and caffeic acid IC_50_ values in the Th-T assay of hIAPP, dihydrocaffeic acid exerted six times higher activity [148].

Human calcitonin, a hormone peptide composed of 32 amino acids produced in the cells of the thyroid gland, is also an amyloidogenic protein. Shen et al. [149] demonstrated the preparation of iron(II, III) oxide (Fe_3_O_4_)-DHCA conjugates nanomaterials and assessed their application as human calcitonin aggregation inhibitors. It has been indicated that Fe_3_O_4_-DHCA nanomaterials inhibited the formation of calcitonin aggregates, and dose-dependent activity was observed in Th-T assays. Moreover, the authors paid attention to the straightforward process of preparation of such nanomaterials and observed their activity even during the presence of oligomers or protofibrils of human calcitonin [149].

Aung et al. [150] presented studies on the effect of selected derivatives of hydroxycinnamic acid on the neutralization of hemorrhagic action induced by snake venom. The most potent compounds were substances bearing an unsaturated double bond and carboxylic functional group; therefore, rosmarinic and caffeic acids, but also sorbic and crotonic acids, exhibited high activity towards the neutralization of hemorrhage. Interestingly, the esters of these compounds, and saturated derivatives such as dihydrocaffeic acid, did not exhibit any activity [150].

The aim of the study of Deng et al. [151] was to shed some light on various tyrosine metabolites as GPR35 (G protein-coupled receptor 35) agonists, which may be potential drugs for diseases associated with abnormal tyrosine metabolism. The authors found that many tyrosine metabolites may be agonists of GPR35. Dihydrocaffeic acid with an EC_50_ value of 342 mM was the ninth compound in the potency rank. Compounds such as 3,3’,5’-triiodothyronine, 3,3’,5-triiodothyronine, and 5,6-dihydroxyindole-2-carboxylic acid, or dihydroxyphenolic acids such as rosmarinic and gentisic acids were definitely of higher effectiveness and potency [151].

## 7. Conclusions

In conclusion, one could ask the question, is dihydrocaffeic acid a phenolic acid similar to any other? Probably not, and the results of the many studies presented above attempt to confirm this statement. DHCA is an extremely valuable compound that occurs in rather small amounts in the natural environment but may appear in fermented food and beverages, as well as being one of the main metabolic products of foods containing caffeic and chlorogenic acids. The interest of the scientific world in this chemical compound has not decreased, as indicated by the research carried out from the 1980s to the present day. Within this review article, comprehensive information about sources, biosynthesis, metabolism and bioavailability, and biological properties, as well as the (chemo)enzymatic modification of dihydrocaffeic acid were provided. The presence of a catechol ring in the chemical structure of this acid extends its food and medical applications. It has been shown that DHCA and its derivatives can be used as food additives with an antioxidant effect, and it has also been demonstrated that, both in vitro and in vivo, they have a protective effect on cells subjected to oxidative stress and inflammation. To sum up, dihydrocaffeic acid and its derivatives, despite some limitations, deserve further study. Before undertaking clinical trials, more comprehensive studies are needed on the use of some promising derivatives or the acid itself, aiming to prove their effectiveness and usefulness in different aspects of life.

## Figures and Tables

**Table 1:** Sources of dihydrocaffeic acid.

Source	Concentration	Units	Reference
leaves of dyer’s woad (*Isatis tinctoria*)	0.03	mg/kg dry weight	[3]
white ray florets of chamomile (*Matricaria recutita*)	NS ^1^	NS	[4]
stems of edible gynura (*Gynura bicolor*)	90.91	mg/g ethyl acetate fraction	[5]
transformed root cultures of *Nepeta teydea*	10.53	mg/kg freeze-dried roots	[6]
the aerial part of asian spicebush (*Lindera glauca*)	0.306	mg/kg dry weight	[7]
the whole grasses of spikemoss (*Selaginella stautoniana*)	2.25	mg/kg fresh weight	[8]
flowers of the Australian rainforest tree (*Polyscias murrayi*)	352.32	mg/kg fresh weight	[9]
concentrated chestnut rose (*Rosa roxburghii*) juice	0.30	g/L	[10]
*Melipona beecheii* Cuban polyfloral honey	NS	NS	[11]
Tantbouchte, Tafizaouine, Tazerzait and Tazizaout varieties of the Algerian ripe date palm fruit (*Phoenix dactylifera*)	NS	NS	[12]
black olive pericarp	1.790 ± 0.030	g/kg dry weight	[13]
black olive brine	0.183 ± 0.001	g/L	
Black Thasos olive cultivar	5–10%	of total phenolics	[14]
Black Conservolia olive cultivar	5–10%	of total phenolics	
Green Douro olive cultivar	12	mg/100 g pulp of fresh olives	
Black Cassanese olive cultivar	3	mg/100 g pulp of fresh olives	
Taggiasca Ligure Italian Extra Virgin Olive Oil	NS	NS	[15]
the red wine Lacrima di Morro d’Alba DOC produced in the region of Marche (Italy)	NS	NS	[16]
Asturian (Spain) natural ciders (*n* = 92)	26.050–147.190	mg/L	[17]
Asturian (Spain) natural ciders (*n* = 8)	55.8–110.5	mg/L	[18]

^1^ The values were not specified.

**Table 2:** Derivatives of dihydrocaffeic acids, their sources and content.

Compound	Source	Concentration	Units	Reference
methyl dihydrocaffeate (**2**)	fresh aerial parts of edible gynura (*Gynura bicolor*)	45.45	mg/kg ethyl acetate fraction	[5]
dihydrocaffeic acid hexose isomers	Romaine, iceberg, and butterhead lettuce (*Lactuca sativa*) cultivars	NS ^1^	NS	[19]
esterified dihydrocaffeic acid with quinic acid	Glasswort (*Salicornia herbacea* L.)	36.6–85.1	mg/100 g dry weight	[20]
Tungtungmadic acid (3-caffeoyl-4-dihydrocaffeoyl quinic acid) (**3**)	Glasswort (*Salicornia herbacea* L.)	8	mg/kg dry weight	[21]
Tungtungmadic acid (3-caffeoyl-4-dihydrocaffeoyl quinic acid) (**3**)	Glasswort (*Salicornia herbacea* L.)	NS	NS	[22]
Tungtungmadic acid (3-caffeoyl-4-dihydrocaffeoyl quinic acid) (**3**)	Glasswort (*Salicornia herbacea* L.)	8	mg/kg dry weight	[23]
Tungtungmadic acid (3-caffeoyl-4-dihydrocaffeoyl quinic acid) (**3**)	Glasswort (*Salicornia herbacea* L.)	0.5125	mg/kg fresh weight	[24]
Salicornate (methyl 4-caffeoyl-3-dihydrocaffeoyl quinate) (**4**)		0.5625		
Podospermic acid (1,3,5-tris(dihydrocaffeoyl)quinic acid) (**5**)	cutleaf vipergrass (*Podospermum laciniatum,* synonym: *Scorzonera laciniata* L.)	358.10	mg/kg fresh weight	[25]
Feruloylpodospermic acid A (1,5-bis(dihydrocaffeoyl)-3-feruloyl quinic acid) (**6**)	the aerial parts of *Scorzonera divaricata*	82.03	mg/kg dry weight	[26]
Feruloylpodospermic acid B (1,4-bis(dihydrocaffeoyl)-3-feruloyl quinic acid) (**7**)	the aerial parts of *S. divaricata*	23.44		
Kukoamine A (N^1^,N^12^-*bis*(dihydrocaffeoyl)spermine) (**8**)	the root bark of Chinese boxthorn (*Lycium chinense*)	NS	NS	[27]
Kukoamine B (N^1^,N^8^-*bis*(dihydrocaffeoyl)spermine) (**9**)	the root bark of Chinese boxthorn (*L. chinense*)	12.07	mg/kg dry weight	[28]
Kukoamine A (N^1^,N^12^-*bis*(dihydrocaffeoyl)spermine) (**8**)	potato (*Solanum tuberosum*) tubers, tomato (*Lycopersicon esculentum*) and *Nicotiana sylvestris*	NS	NS	[29]
N^1^,N^8^-*bis*(dihydrocaffeoyl)spermidine (**10**)		NS	NS	
N^1^,N^4^,N^12^-*tris*(dihydrocaffeoyl)spermine (**11**)		NS	NS	
N^1^,N^4^,N^8^-*tris*(dihydrocaffeoyl)spermidine (**12**)		NS	NS	
Kukoamine A (N^1^,N^12^-*bis*(dihydrocaffeoyl)spermine) (**8**)	the stems of Enoki (*Flammulina velutipes*)	NS	NS	[30]
N^1^,N^8^-*bis*(dihydrocaffeoyl)spermidine (**10**)	bitter fraction of Lulo (*Solanum quitoense* Lam.)	67.2	mg/kg	[31]
N^1^,N^4^,N^8^-*tris*(dihydrocaffeoyl)spermidine (**12**)		100.8	mg/kg	
N^1^,N^4^,N^8^-*tris*(dihydrocaffeoyl)spermidine (**12**)	fruit pulp of Lulo	1.6	mg/kg	
	freeze-dried Lulo fruits	25.1	mg/kg	
	spray-dried Lulo fruits	25.0	mg/kg	
(3α,21β)-Lycophlegmariol A (**13**)	the methanol extract of common tassel fern (*Lycopodium phlegmaria* L.)	4.54	mg/kg dry weight	[32]
(3β,21β)-Lycophlegmariol B (**14**)		5.17		
(3β,21α)-Lycophlegmariol D (**15**)		29.54		
(3β,21α)-Lycophlegmarin (**16**)		2.64		
(3α,21β)-Lycophlegmariol A (**13**)	the aerial parts of *Huperzia phlegmaria* (*L. phlegmaria* L.)	10.00	mg/kg dry weight	[33]

^1^ The values were not specified.

**Table 3:** Anticancer activity of dihydrocaffeic acid and its derivatives on different cancer cell lines.

Compound	Cancer Cell Line	IC_50_ (mM) *	Reference
methyl dihydrocaffeate (**2**)	mouse leukemia cell line L1210	0.024	[108]
ethyl dihydrocaffeate (**17**)		0.023	
isopropyl dihydrocaffeate (**45**)		0.019	
butyl dihydrocaffeate (**19**)		0.020	
*tert*-butyl dihydrocaffeate (**46**)		0.024	
hexyl dihydrocaffeate (**20**)		0.009	
octyl dihydrocaffeate (**21**)		0.009	
benzyl dihydrocaffeate (**47**)		0.022	
phenethyl dihydrocaffeate (**25**)		0.012	
3-phenylpropyl dihydrocaffeate (**48**)		0.010	
methyl dihydrocaffeate (**2**)	breast cancer cell line MCF-7	0.115	
ethyl dihydrocaffeate (**17**)		0.105	
isopropyl dihydrocaffeate (**45**)		0.069	
butyl dihydrocaffeate (**19**)		0.110	
*tert*-butyl dihydrocaffeate (**46**)		0.050	
hexyl dihydrocaffeate (**20**)		0.105	
octyl dihydrocaffeate (**21**)		0.060	
benzyl dihydrocaffeate (**47**)		0.120	
phenethyl dihydrocaffeate (**25**)		0.132	
3-phenylpropyl dihydrocaffeate (**48**)		0.129	
dihydrocaffeic acid (**1**)	human melanoma cell line SK-MEL-24	2.200	[110]
dihydrocaffeic acid (**1**)	leukemic cell line U-937	>2.000	[111]

* The concentration that decreases cell viability by 50%.

## Data Availability

The data presented in this study are available on request from the corresponding author.
